# The state of the art in beyond 5G distributed massive multiple-input multiple-output communication system solutions

**DOI:** 10.12688/openreseurope.14501.1

**Published:** 2022-09-02

**Authors:** E. Meyer, D. Kruglov, M. Krivic, M. Tanveer, R. Argaez-Ramirez, Y. Zhang, A. Briseno Ojeda, K. Smirnova, K. Alekseev, M. Safari Mugisho, B. Cimbili, N. Farid, Y. Dang, M. Shahid, M. Ensan, J. Banar, H. Bao, M. Matters-Kammerer, U. Gustavsson, F. Demuynck, T. Zwick, M. Acar, C. Fager, M. van der Heijden, M. Ivashina, D. Caratelli, M. Hasselblad, C. Ulusoy, A.B. Smolders, K. Eriksson, M. Johannson, R. Maaskant, R. Quay, D. Floriot, M. Bao, L.A. Bronckers, J. Fridén, M.C. van Beurden, B.P. de Hon, C. Kolitsidas, D. Blanco, F.M.J. Willems, T. Eriksson, A. Filippi, F. Ponzini, U. Johannsen

**Affiliations:** 1 Eindhoven University of Technology, Den Dolech 2, 5612 AZ Eindhoven, The Netherlands; 2 Chalmers University of Technology, Chalmersplatsen 4, 412 96 Göteborg, Sweden; 3 Keysight Technologies, Kortrijksesteenweg 1093B, 9051 Gent, Belgium; 4 Karlsruhe Institute of Technology, 6131 Karlsruhe, Germany; 5 Fraunhofer Institute for Applied Solid State Physics, IAF, Tullastraße 72, 79108 Freiburg, Germany; 6 Ericsson AB, Lindholmspiren 11, 417 56 Göteborg, Sweden; 7 NXP Semiconductors, High Tech Campus 60, 5656 AG Eindhoven, The Netherlands; 8 The Antenna Company, High Tech Campus 29, 5656 AE Eindhoven, The Netherlands; 9 Gapwaves, Nellickevagen 22, 412 63 Gothenburg, Sweden; 10 United Monolithic Semiconductors SAS, Bâtiment Charmille, Mosaic parc de Courtaboeuf, 10 avenue du Québec, 91140, Villebon-sur-Yvette, France; 11 Ericsson Telecomunicazioni SpA, Via Anagnina 203, 00118 Rome, Italy

**Keywords:** Millimetre-wave communication, MIMO, Antennas-in-Package, System-in-Package, beyond-5G

## Abstract

Beyond fifth generation (5G) communication systems aim towards data rates in the tera bits per second range, with improved and flexible coverage options, introducing many new technological challenges in the fields of network architecture, signal pro- cessing, and radio frequency front-ends. One option is to move towards cell-free, or distributed massive Multiple-Input Multiple-Output (MIMO) network architectures and highly integrated front-end solutions. This paper presents an outlook on be- yond 5G distributed massive MIMO communication systems, the signal processing, characterisation and simulation challenges, and an overview of the state of the art in millimetre wave antennas and electronics.

## 1 Introduction

Fifth generation (5G) networks aim to achieve a thousandfold data rate improvement with respect to the capability of fourth generation (4G) networks. From an engineering point of view, this goal requires an increase in the frequency bandwidth of operation for higher channel capacity and the number of antennas per base station for improved spectral efficiency ^1^. Distributed Massive Multiple-Input Multiple-Output (DM-MIMO), discussed in Section 2, is considered the most promising concept for future communication networks. Its distributed nature, large number of up- and downlink channels, and increased path loss in mmWave range pose serious problems for adequate signal processing. Section 3 outlines the current state-of-the-art Distributed MIMO techniques and architectures, and discusses the multi-physics simulation issues rising from the growing scale of the problem.

For a MIMO system to work, the base stations must have versatile beamforming capabilities. In practice, this means large number of antennas closely packed in arrays, where each antenna is connected to a corresponding channel in the beamforming integrated circuit (IC). Section 4 describes the most prominent issues in antenna design at higher frequencies and state-of-art solutions. Transceiver ICs must produce enough power, while being as efficient as possible, since the losses scale with the number of array elements. A transceiver chain has to be linear, have high-gain, high output power, and a wide frequency band. It is impossible to maximize all these parameters simultaneously, and so trade-offs are made specific to application and use case. Several challenges also exist on a component specific level, therefore Section 5 outlines the state-of-art, challenges and requirements for the mmWave electronics in future communication networks.

## 2 Distributed massive MIMO

Over the past few decades, increasing the number of access points (APs) and antennas per AP has been an enabling factor to increase the data rate in wireless cellular networks. This approach would result in having smaller cells often with massive arrays of receiving/transmitting antennas to take advantage of the spatial reuse of the spectrum ^2^. Employing massive array of antennas per AP not only increases the high sum spectral efficiency but also leads to simultaneously providing good service to many user equipments (UEs) ^3^.

A conventional wireless network is typically divided into cell regions, and categorizes UEs into the cell-center and cell-edge classes. These two subsets of UEs could experience different path losses leading to varied quality of service in the network. This problem has been addressed in the distributed massive MIMO (DM-MIMO) networks where a large number of APs are located across the service area and simultaneously provide service for the UEs. Therefore, UEs would benefit from a more uniform quality of service due to the diversity in the experienced pathloss from different APs. The cooperation among APs discards the cellular structure in this distributed network. As a result, in the literature, this network is also referred to as cell-free massive MIMO ^4^. In a DM-MIMO network, APs in a service area are connected to a central processing unit (CPU) through a network of fronthaul links. In general, CPU in this network provides data for the APs and ensures cooperation and synchronization among them.

Distributed beamforming holds the potential for enhancing energy efficiency to a great extent in wireless networks. MIMO techniques have proved to be remarkable in enhancing both the capacity and energy efficiency of a wireless communication system and is a key technology enabler in current mobile wireless networks ^5, 6^. However, because all the antennas are co-located the line of sight (LOS) conditions between base station and user cause high correlation among various users and proves to be a bottleneck for a system throughout ^5, 6^. An alternate method is to increase the spatial degrees of freedom of each MIMO channel by making use of a concept called distributed. Distributed MIMO simultaneously achieves suppression of multiuser interference via spatial decoding and dense coverage such that a subset of the antennas is always present close to the user ^5– 7^.

The promising performance metrics of massive MIMO such as power efficiency and spectral efficiency can only be realized to maximum extent if it is operated in rich scattering environments i.e. each of the channels from a base station to a single user are significantly different ^7^. Thus, distribution of the antennas over a cell proves to be a straightforward solution to achieve low channel correlation. Ideally, a user being serviced simultaneously by multiple front ends will always have LOS conditions with at least one of them, while the network would be adaptive enough to respond to the user. Among others, benefits in favour of this topology are low latency, high throughput, and diversity of applications that can be serviced ^7^.

### 2.1 Architectures of distributed MIMO

**Figure 1.**
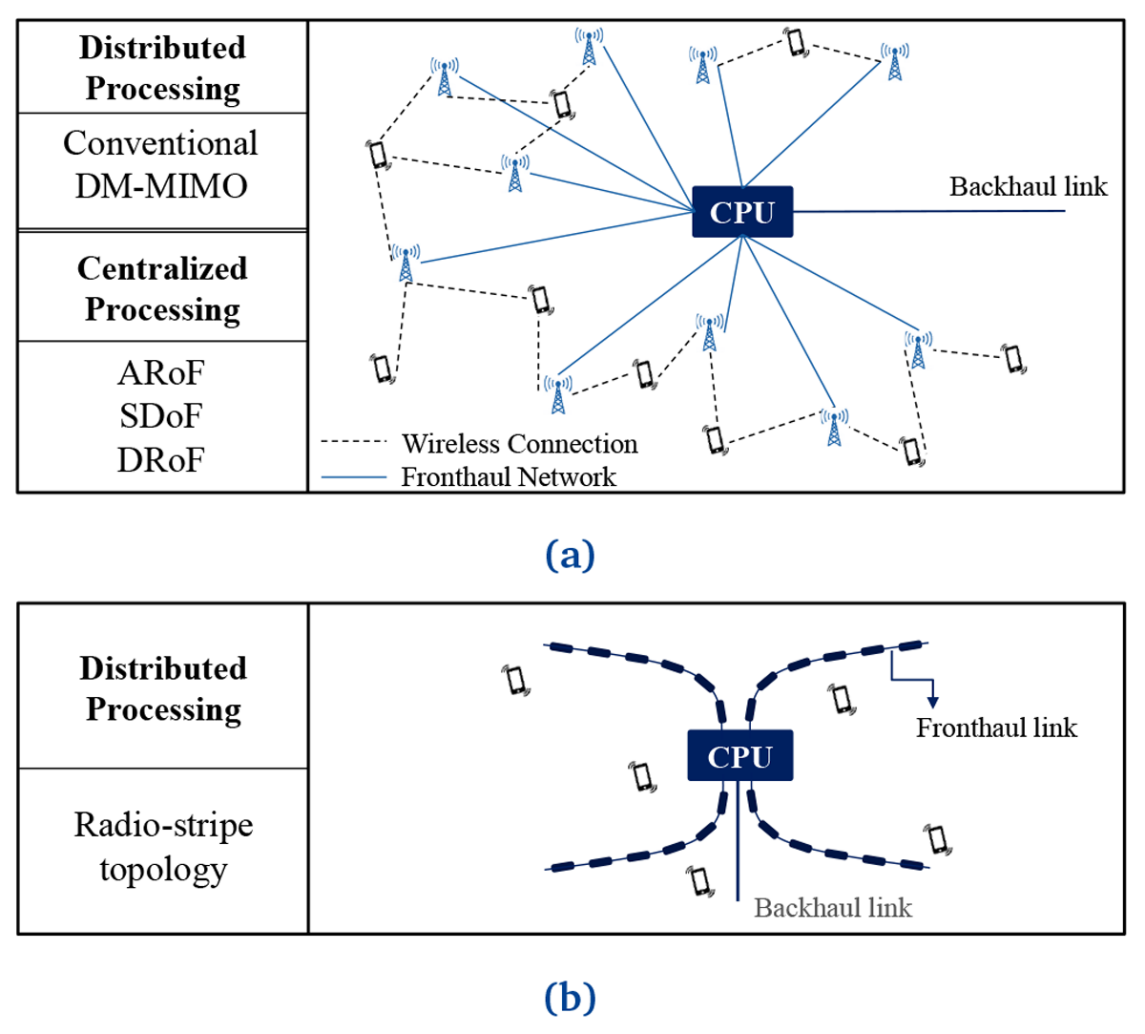
An overview of different processing approaches. Conventional Distributed Massive Multiple-Input Multiple-Output (DM-MIMO) networks such as analog radio, radio frequency or digital over fiber (ARoF, SDoF and DRoF respectfully) in ( **a**). Radio-stripes topology in ( **b**).

In the last couple of years, various kinds of DM-MIMO architectures have been proposed and in few cases implemented to demonstrate proof of concept ^5– 10^. Speaking from point of view of how signal processing component of MIMO is implemented, these architectures can be broadly classified into two categories, distributed and centralized signal processing, as shown in Figure 1.

In distributed signal processing, both CPU and APs are involved in signal processing and communicate with each other via cables or optical fibers ^5, 6, 10^. In these architectures common clock or timing synchronisation is often provided via IEEE 1588 protocol or more recently white rabbit scheme ^5^.

Besides the general form of DM-MIMO implementation (see Figure 1(a)), radio stripe architecture (see Figure 1(b)) was proposed for dense scenarios, e.g., stadiums, stations and malls, by Ericsson in Mobile Congress 2019 ^11^. In radio stripes, multiple APs share one fronthaul cable for synchronisation, data transmission and power supply. The APs located in each of the stripes form a line network. These line networks can also be seen between some of the APs and the CPU in the general form of the DM-MIMO network. This architecture falls under the category of distributed processing DM-MIMO in the sense that each access point is supposed to have dedicated circuitry for digital signal processing (DSP) and the analog-to-digital or digital-to-analog converters (ADC/DAC) implemented ^10^. It also requires common timing signals to be distributed in a similar way and suffers from the same limitation of not having common local reference frequency for the distributed front ends. However, from logistic point of view it is much more feasible to implement, as it requires only one cable from CPU to attach to many APs. Multiplexing/demultiplexing additionally needs to be performed at both the CPU and access point to make this topology successful.

The other architecture is classified as centralized signal processing for DM-MIMO – also see Figure 1. In this category of architectures ^8, 9^, all the signal processing is done at CPU while the APs consist of just the Radio Frequency (RF) front ends. No timing synchronisation is required in this case as the center of all APs is located at the CPU. Frequency synchronisation in this case is applicable only when the front ends at APs consist of some upconversion to higher frequencies. In the absence of an upconversion stage at the APs, a common local oscillator (LO) is distributed by the CPU. Electrical to optical and optical to electrical converters are used respectively on either side of fiber. The AP only consists of RF front ends that consist of power amplifiers, low noise amplifiers, antennas, switches, and filters.

**Figure 2.**
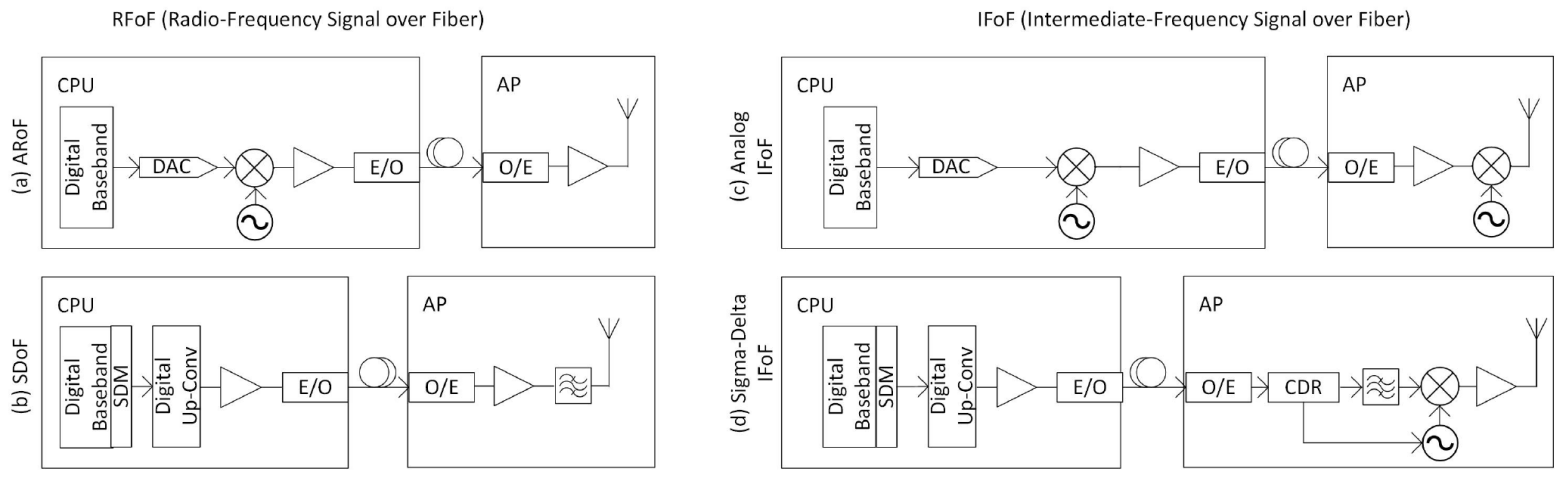
Analogue Radio over Fiber (ARoF) and Sigma-Delta over Fiber (SDoF) architectures. CDR, Clock Data Recovery; AP, Access Point; DAC, Digital Analog Converter; CPU, Central Processing Unit; IFoF, Intermediate-Frequency signal over Fiber; SDM, Sigma-Delta Modulation.

One of the topologies to implement central processing distributed MIMO is analogue radio over fiber (ARoF) ^8^. In 12, radio frequency over fiber (RFoF) and intermediate frequency over fiber (IFoF) are shown for analogue radio solutions in Figure 2 (a) and (c) respectively. Analog radio receives analog signal from fiber as keeping digital to analog converter (DAC) at CPU. Occasionally, the ARoF architecture can also include mixers and local oscillators to transmit at higher frequencies. ARoF is advantageous for its optical spectral efficiency and simple remote radio unit architectures ^12^. However, it has a drawback to being sensitive to nonlinear distortions and fiber transmission impairments that can cause large phase incoherency between the channels, especially if the frequency carrying the data in the optical fiber is high. Thus, this can lead to a more frequent need of performing calibration that would degrade system data throughput.

A combination of analog radio over and digital radio over fiber architectures called sigma-delta over fiber (SDoF) ^9^ has recently been demonstrated as another central processing DM-MIMO topology. In this case, the AP contains minimal components. The digital data at the CPU is not converted to analogue data but gets up-converted in the digital domain and then modulate the optical frequency, which at the other end after being demodulated is passed through a bandpass filter to recover the radio frequency (RF) passband frequency as shown in Figure 2 (b). This topology combines the advantages of digital radio over fiber in that it has much less sensitivity to non-linearities and distortion. However, the SDoF topology has a limitation of being employed at lower frequency bands. For frequencies 28 GHz and above, additional mixers would be needed at each of the access points which would necessitate either common LO distribution architecture to be implemented or to carry out synchronization algorithms similar to architectures employed for distributed processing DM-MIMO; thus losing on the advantages that were gained in ARoF topology. Additionally, transmitter oversampling is proportional to RF therefore not suitable for FR2 or above frequency bands. Figure 2 (d) shows the state-of-the-art design for mmWave SDoF topology.

**Figure 3.**
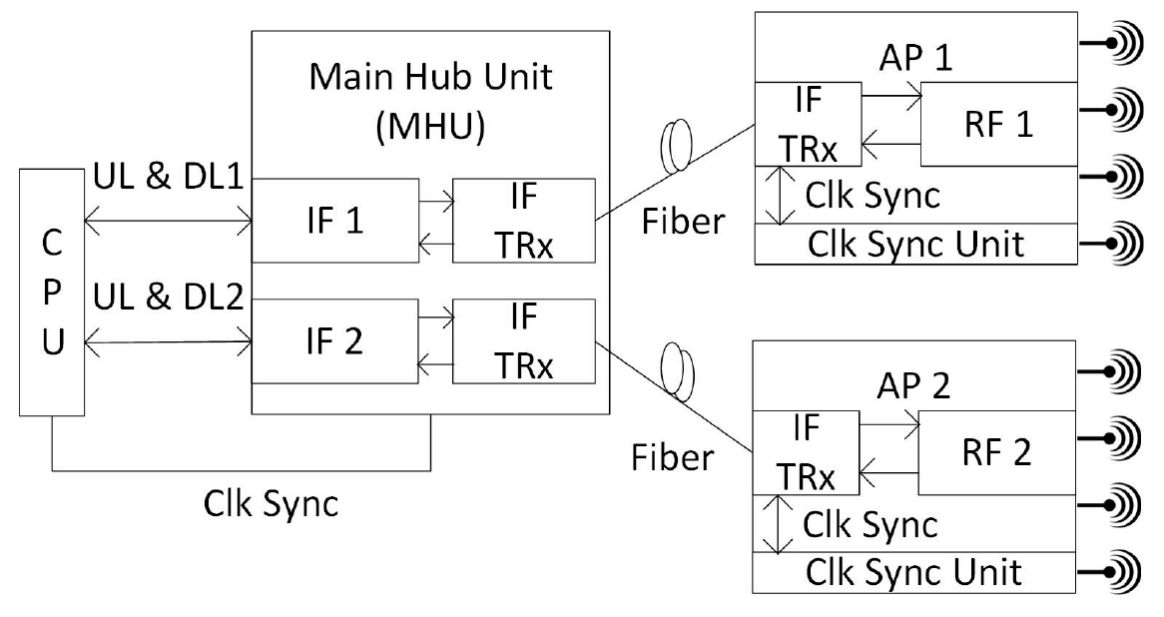
Distributed Radio over Fiber (RoF) Multiple-Input Multiple-Output (MIMO) system. CPU, Central Processing Unit; TRx, Transceivers; AP, Access Point; IF, Intermediate Frequency; UL, Uplink; DL, Downlink.

Besides the above discussion, a distributed 4-by-4 MIMO was demonstrated as a state-of-the-art mmWave communication radio design as in Figure 3. This system supports 1 GHz bandwidth for 1 km distance and also commercially worked for PyeongChang 2018 Olympic Winter Games ^13^. In the CPU, an intermediate signal is generated for downlink communication. Then, the main hub unit (MHU) combines it with the clock and management (C&M) signal in a dedicated frame structure to transmit it through fiber. The transceivers (TRx) receives the optical signal for the AP intermediate frequency (IF) unit which does signal processing and upconversion to get the mmWave signal. Finally, the array antenna radiates wireless signal into the open air. C&M signal helps to synchronize radios for MIMO applications.

In summary, distributed processing architecture does clock or timing synchronization by following the standard protocol as being widely used in industry. In comparison, centralized processing architecture simplifies synchronization in general, and becomes a hot topic in the research area. Furthermore, in centralized processing architecture, ARoF has the simplest radio structure while RoF is the most complex solution; SDoF does not need DAC by implementing one-bit digital signal in the algorithm.

## 3 Signal processing for distributed massive MIMO

### 3.1 Synchronization and calibration

In a communication system, the receiver must be synchronized with the incoming signal to work correctly, and its performance is related to the accuracy of the synchronization. Synchronization includes timing synchronization and carrier synchronization between the receiver and the incoming signal. Timing synchronization is the receiver’s procedure for determining the time instants for sampling the incoming signal. Carrier synchronization is the receiver’s procedure to adapt the local oscillator’s carrier frequency and phase with those of the received signal. Generally, there is no prior knowledge about the transmitted signal related to the physical wireless channel or propagation delay for a wireless receiver. Furthermore, low-cost oscillators typically used in communication receivers have some drift.

Combining the benefits of "massive MIMO" and "small cells" will result in a wireless network architecture named DM-MIMO. In 6, a cost-effective scenario is expressed that included inexpensive APs connected to the CPU via a conventional wired digital backbone. An accurate, common clock for synchronizing the APs is needed using over-the-air signaling or a wired backhaul network.

In the context of an uplink-pilot/downlink-precoded transmission cycle, each AP individually processes the observed uplink pilots transmitted by UEs through a synchronization block. Each AP estimates the uplink channel and communicates this to the CPU through a digital backhaul network. The CPU is, therefore, able to calculate the DM-MIMO precoding matrix. In the downlink transmission phase, the precoded signal at each AP is calibrated. The calibration step compensates for the mismatches caused by the transmitter and receiver AP hardware, such as the non-reciprocal amplitude scaling and phase rotations. On the same time-frequency slot, all the APs in the DM-MIMO network send data simultaneously.

Synchronization is performed for uplink and downlink operations. The synchronization block in the AP synchronizes the frame and carrier frequency to transmit and receive on the assigned time slots. Jointly precoded APs send simultaneously downlink data that passed the synchronization block to the UEs. In this AP block, the timing misalignment and the relative phase rotation of the downlink signal are compensated through some signal processing algorithms. These algorithms estimate the received signal parameters affected by a timing offset and carrier frequency offset to compensate them.

Due to the Time Division Duplex (TDD) used in massive-MIMO systems, the physical propagation channel can be approximated as reciprocal, which means the same channel is used in both the uplink pilot slot and the downlink data slot. Then, it is feasible to attain the downlink Channel State Information (CSI) via channel reciprocity based on the uplink pilots. While, the propagation channel between the transmitter and receiver’s antenna is reciprocal, the hardware is not reciprocal, and there are an unknown amplitude scaling and phase shift between the uplink and downlink channels. A built-in self-calibration capability does not exist in the typical commercial grade radios, and nonreciprocity between the receiver and the transmitter hardware is rectified explicitly via a TDD reciprocity calibration protocol.

Calibration is used to compensate for the difference between uplink and downlink RF front-end components. In centralized massive-MIMO, antennas are placed near each other, and additional circuits can provide the reciprocity calibration. But there are two methods for reciprocity calibration in distributed massive-MIMO: full calibration and partial calibration ^14^. In full calibration, an AP can determine its own mismatch matrix and the user equipment (UE) mismatch matrix using the downlink CSI feedback from the UE ^6^. In partial calibration, only the AP mismatch matrix is determined to achieve multi-user interference suppression. The UE mismatch matrix effect is considered negligible due to its little impact on functional systems performance. This calibration method can be planned besides synchronization ^15^.

### 3.2 Signal compression and quantization

In practice, the fronthaul links connecting APs and the CPU typically have limited capacity which impacts the spectral efficiency in a DM-MIMO network. Therefore, performing signal compression techniques on the fronthaul load becomes a necessity. In general, APs’ uplink observations are independently compressed and transmitted to the CPU over the capacitated fronthaul links. The CPU decompresses the received signals from each AP separately, and then performs joint decoding of UEs messages. The compression codebook at each AP is determined based on the AP’s channel state information. Taking into the account that APs’ observations are correlated, it is possible to further reduce the fronthaul rate by exploiting this correlation using distributed Wyner-Ziv compression. In this compression scheme, each AP will be required to have knowledge of the joint statistics of observations across all APs in order to determine the optimal joint compression codebook. Consequently, decompression and decoding will be performed jointly for all APs’ relayed signals at the CPU. Choosing the optimal joint compression codebook for all APs is challenging and the fact that statistics in a wireless network change rapidly makes this compression approach rather impractical ^16^. A more practical compression method is proposed in 17 for scenarios with imperfect statistical knowledge of the correlation among APs’ observations. The compression problem is formulated based on a deterministic worst-case scenario and the solution is found by solving Karush-Kuhn-Tucker conditions. Numerical results in 17 have shown that sizeable errors in the statistical information at the APs are tolerable and would not severely affect the distributed compression performance among APs.

In some distributed architectures, including radio-stripes, APs are fairly simple units consisting of antennas and circuit-mounted chips while the CPU is responsible for the baseband processing ^11^. These structures are addressed in the literature of oblivious relaying where APs operate without knowledge of UEs codebooks. Randomized encoding is used in the literature to model the communication over the wireless links (to take into the account the lack of codebook knowledge at the APs) and a type of compress-and-forward scheme including distributed Wyner-Ziv compression is implemented throughout the fronthaul links ^18– 20^. Among other compression techniques are decode-and-forward ^21^ and compute-and forward ^22^ where APs are constrained to have knowledge of the codebooks employed by UEs. The rates provided by these schemes can sometimes be higher than the ones based on oblivious processing. Although, as the number of APs and UEs increases in the network, the signaling overhead to provide knowledge of UEs codebooks at the APs and performing encoding/decoding can become excessive.

### 3.3 Precoding and channel estimation

**Figure 4.**
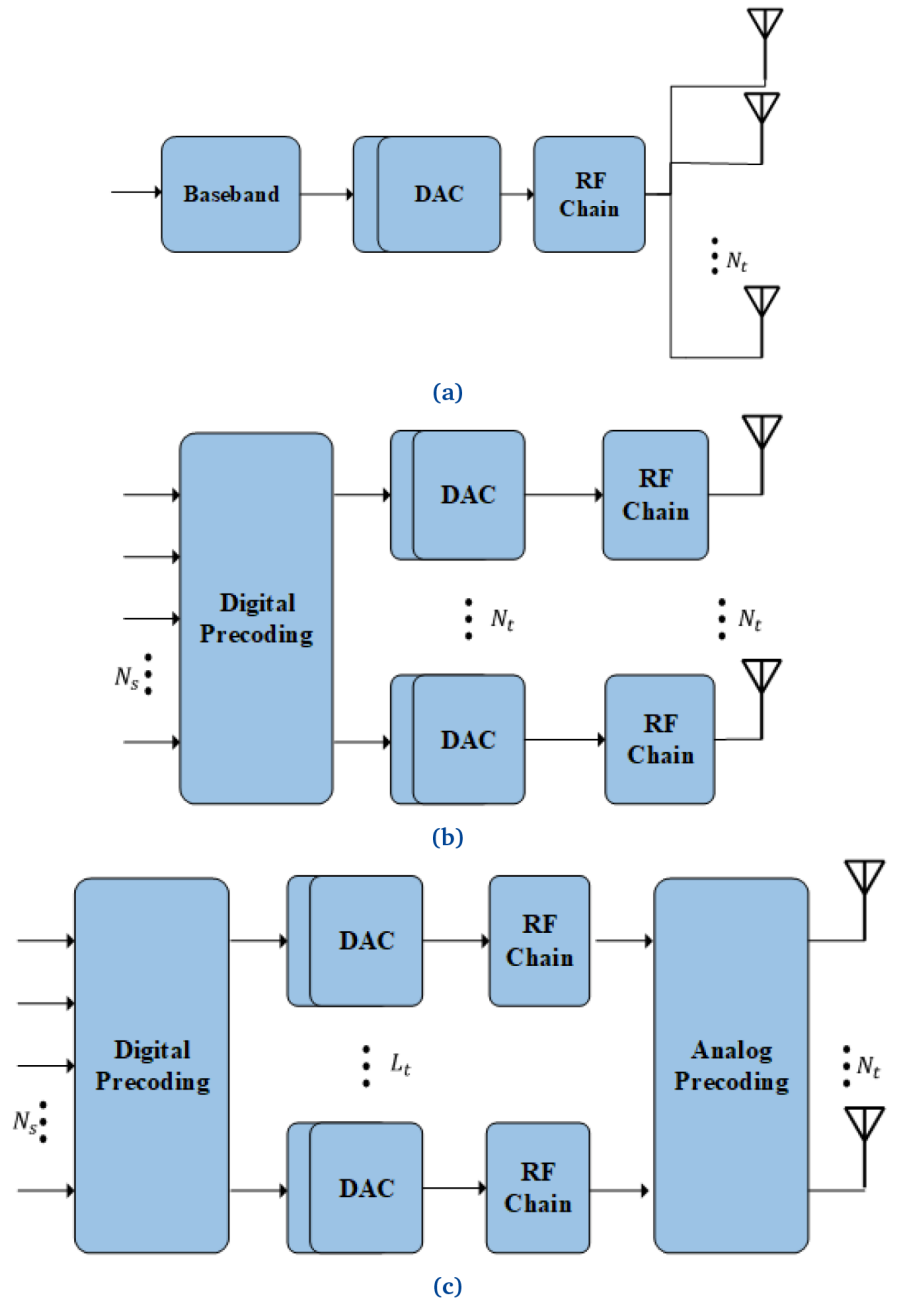
Precoding architectures: ( **a**) Analog, ( **b**) Digital and ( **c**) Hybrid. With *N _s_* data streams, *N _t_* total number of antennas and *L _t_* << *N _t_*. DAC, Digital Analog Converter; RF, Radio Frequency.

The design of precoding schemes is highly essential for mmWave massive MIMO cellular systems. Precoders optimize the network’s performance using interface cancellation in advance, controlling the original signal’s phase and/or amplitude. This process is also known as beamforming. The primary purpose of this procedure is to transmit highly directional beams pointing to the terminals with minimal or no interference. The selection of the precoding scheme affects the signal processing design and the performance of MIMO systems. The precoding schemes can be classified into three ( Figure 4):

*Analogue precoding:* In this scheme, only one RF chain is employed to transmit a single data stream, leading to relatively low hardware cost and energy consumption. The analog circuit is used to adjust the properties of the signals to achieve an array gain. However, since the analog circuit uses phase shifters to adjust the phase of the signal, as shown in Figure 4(a), their implementation at mmWave frequencies incurs higher loss. Moreover, an extension of this scheme to multiuser systems seems not trivial ^23^, and so it cannot be used for mmWave massive MIMO networks. Nevertheless, this scheme has been adopted in indoor mmWave communications, such as 60 GHz WLAN ^24^.

*Digital precoding:* this precoding is performed at baseband, where each antenna requires a dedicated RF chain ^25^. Hence, it can be employed in both single-user and multiuser systems. The scheme has been used by conventional MIMO systems, but becomes unrealistic for mmWave massive MIMO systems. Firstly, the high resolution ADC/DAC needed are extremely power hungry. Secondly, this architecture requires *N _t_* copies of the entire radio front-end, as shown in Figure 4(b), increasing the power consumption and manufacturing cost. Therefore, although the fully digital architecture for mmWave massive MIMO systems might be available in the future, alternative schemes are required for emerging mobile networks.

*Hybrid Precoding:* the architecture depicted in Figure 4(c) was agreed to be employed in 5G systems at the 3GPP RAN1 meeting ^26^. It can be considered an extension of the analog architecture to the multi-stream scenario and can significantly reduce the number of RF chains. The main idea is to divide the conventional digital signal processing of large size into two parts: an analog signal processing stage (realized by the analog circuit) and a dimensional reduced digital signal processing (requiring a small number of RF chains). The hybrid precoding design is known to improve the energy efficiency (EE) of the system ^27, 28^. The analog circuits in this scheme can be implemented in different circuit topologies, such as the fully-connected, partially-connected, and lens antenna array ^25, 29, 30^. The various implementations of analog circuits lead to different hardware constraints.

*Channel state information (CSI)* is essential to leverage the antenna gain provided by multiple antennas specially on mobile communications where the channel changes rapidly. With a conventional digital scheme at the receiver, the use of pilots to acquire CSI is the most common technique to use. For hybrid architectures, on which the signal processing is split into and analog and digital part, the former is use to enhance the signal power and the latter is design to suppress inter-user interference ^31– 33^. However, the CE is rather complicated since the information on each antenna cannot be extracted simultaneously. Additionally, as the dimension of the channel matrix increases, in the context of mmWave frequencies, the operations required to perform the CSI acquisition involve higher computational complexity. To cope with this problem, one of the techniques recently used is the exploitation of spatially sparsity and temporal correlation of massive MIMO channels. Nevertheless, it is still difficult to design efficient algorithms compared to conventional MIMO systems given the additional hardware constraints that the use of a hybrid scheme brings. That is why, the joint design of CE and precoding/combining algorithms, as well as network architecture tailored to mmWave massive MIMO systems is still an open research topic.

### 3.4 Multiple access techniques

One key component in communication systems is the multiple access technique that it employs.

*Non-Orthogonal Multiple Access (NOMA):* in this scheme, the time-frequency resources of each user are allocated based on two regimes: power-based and code-based. Recent research has shown the potential of this technique in combination with mmWave massive MIMO systems implementing hybrid precoding. MIMO-NOMA reaches a better performance in terms of achievable sum-rate and robustness when compared with conventional MIMO systems. The main idea is that one beam can support several users with the help of intra-beam superposition coding and successive interference cancellation (SIC) ^34, 35^. Therefore, the challenge is to design optimum power allocation algorithms that can achieve a higher sum-rate.

**Figure 5.**
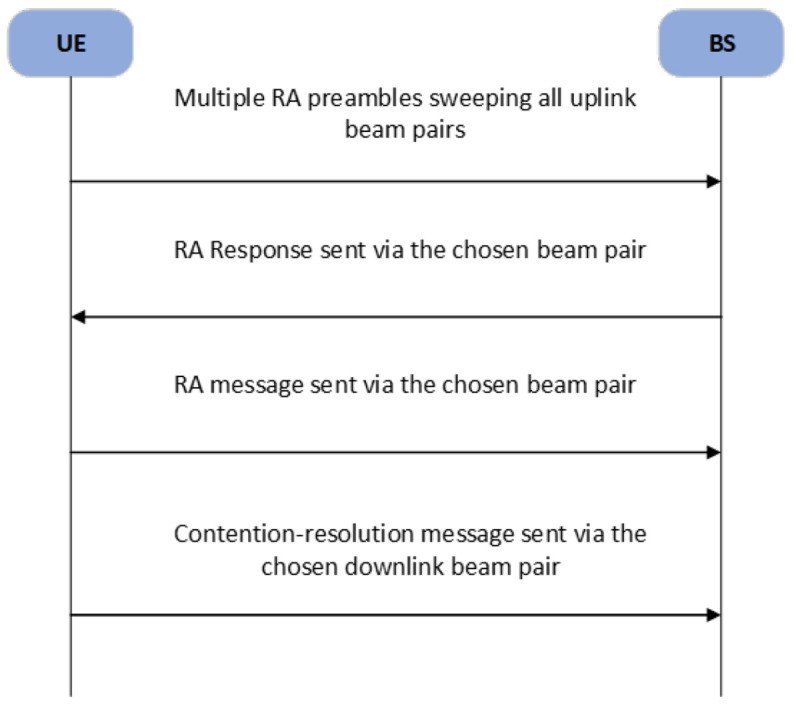
The Random Access (RA) procedure in mmWave beamforming cellular networks. BS, Base Station; UE, User Equipment.

*Random Access (RA):* to fully exploit the beamforming gain, the beam direction and the channel path of both the user and base station (BS) must be aligned. Using the beam quality estimation and information exchange between the UE and BS, the beam can be successfully aligned. A random access procedure in mmWave cellular networks is illustrated in Figure 5. However, there are certain moments when the best beam direction cannot be known a priori, as is the case of initial access. Most initial techniques can be classified into six groups: exhaustive search, iterative search, statistical, meta-heuristics, context-information based, and machine learning. All these techniques attain to search for the best beam pair between the user and the BS. The last four strategies mentioned also decreased the delay caused by searching in all directions, such as their counterparts exhaustive and iterative research. This delay must be small to meet some requirements for low end-to-end latency in subsequent communication systems. Initial access is still considered an open issue ^36^.

### 3.5 Channel emulation for OTA verification

Performance of DM-MIMO highly depends on the characteristics of the propagation channel. Higher path loss, atmospheric attenuation, rain-induced fading, foliage attenuation, material penetration loss, and ease of blockage are some of the propagation challenges when migrating from microwave to mmWave frequencies ^37, 38^. Specifically, the electromagnetic waves will undergo less diffraction implying increased losses due to blocking which will result in a sparse multipath channel. Furthermore, the predicted outline for mmWave applications covers a wide range of user scenarios, namely indoor environments, outdoor environments, indoor-to-outdoor scenarios, and high mobility scenarios ^39– 43^. This translates into diverse propagation channels that differ vastly from each other in terms of power delay profile, delay spread, Doppler spread, and power angle profile. With decreased patchy coverage each use case is more specific than at lower frequencies where diffraction smears out the angular power profile. The device performance will vary more between use cases and even between actual installations, hence, evaluating the performance under realistic channel conditions with controlled angular profile is of great importance.

Considering the small component size and high integration of antenna systems as well as the large number of antenna elements, performing conducted tests is not feasible at mmWaves ^44^. To carry out reliable and repeatable Over-The-Air (OTA) measurements, the behavior of the propagation channel should be emulated in a controlled laboratory environment. Anechoic Chambers (AC) and Reverberation Chambers (RC) are two test facilities widely used for this aim. By utilizing Multi-Probe Anechoic Chamber (MPAC) ^45, 46^ and Radiated Two-Stage (RTS) ^47– 49^ methods, OTA measurements are executed inside an AC. Limitations of the aforementioned methods are that they require a large number of range antennas at mmWaves, the calibration procedure is complex and careful antenna characterization must be performed. The RC offers time-efficient, cost-effective, and more flexible measurement setup where a rich multipath environment with different Rician K-factors can be obtained ^50, 51^. The main challenge in this regard is introducing the lost angular dependencies which is the topic of ongoing research ^52, 53^. An ideal AC can be considered as a Gaussian channel with a line-of-sight link while an ideal RC imitates a Rayleigh channel and is spatially white. A real-life scenario is most probably in between these two corner cases. Considering the multitude of use cases with unique channel characteristics, a need for channel emulation platform with capability to adapt to the desired scenario is foreseen.

### 3.6 Multi-physics simulation of MIMO systems

**Figure 6.**
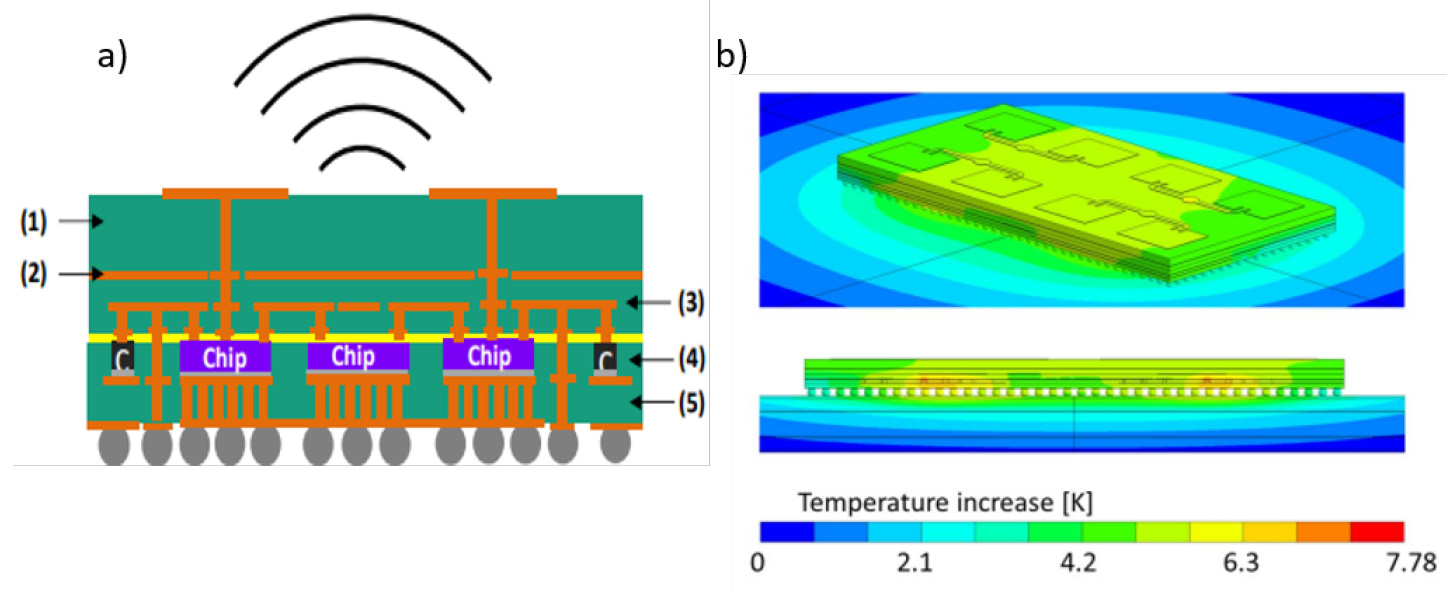
**a**) Example of Antenna-in-Package (AiP) for 5G mmWave side view (left) and top view (right) 1) antenna layer; (2) shielding layer; (3) re-distribution/routing layer; (4) component layer; (5) temperature control layer; **b**) Example (AiP) showing simulation result of temperature distribution/increase due to 1W of power loss. ©2020 IEEE. Reprinted, with permission, from 57.

Multi-physics simulation can play an important role in the design and development of MIMO hardware architectures. This model technique can help to predict thermal and electrical properties in these complex multi-physics system thereby help in predicting thermal stress due to high temperature gradients and dissimilar thermal expansion coefficients which may lead to mechanical failures (e.g. delamination and lift-off ^54^) and change in the EM properties of the materials which in turn affects signal and power integrity issues, such as clock skew, unintentional voltage drops, and spectrum shifts for filters and resonators ^55^. In this way this advanced numerical method helps us to provide better design solutions with regards to thermal management and improved performance of communication systems. One such example as shown in Figure 6b demonstrates simulation result showing temperature distribution across different layers of AiP given in Figure 6a.

It is therefore important to understand the resulting multi-physical modeling technique, its associated challenges and possible bottlenecks for the design and analysis of such integrated communications systems.

***3.6.1 Numerical modelling and simulation*.** In the past few decades the development of numerical experimentation (numerical modelling and simulation method) as a powerful research tool ^56^ proved to be a very effective technique in modelling multi-physics phenomena across multiple time and length scales associated with electro-thermo-mechanical design of the present complex system. 

With this powerful tool enabled by a recent advancement in the computing technology, it is now possible to develop models that can help to test the designs of hardware architecture (from antenna to base station) under different operating conditions required to meet the 5G challenges. It can thus help in rapidly prototyping and optimizing these designs before fabrications and thereby reduces cost and increases reliability, scalability and increases development speed. It is worth mentioning here that although there are various numerical methods ^56^ that are quite similar in representing a systematic numerical method for solving partial differential equation (PDE), they differ however, in implementation, robustness, accuracy and speed. Among these the finite element method (FEM) has gained more popularity in the sense that it is a very general method i.e. solving the resulting system of equations is very similar to well-known and efficient methods used for structural and electromagnetic analysis. It has the capability to locally approximate the physics field very accurately just by increasing the order of polynomial of elements. Moreover, the ability of combining different kinds of physical quantities (e.g. electromagnetic field, mechanical and heat transfer) within each element also called mixed formulations and ability to simulate more complex structures makes it particularly important for multi-physics analysis ^58^.

***3.6.2 Multi-physics modelling*.** Multi-physics model combined with co-simulation method helps to predict and capture the electro-thermal, thermo-mechanical, and electro-mechanical interactions at chip package and package-system interface.

**Figure 7.**
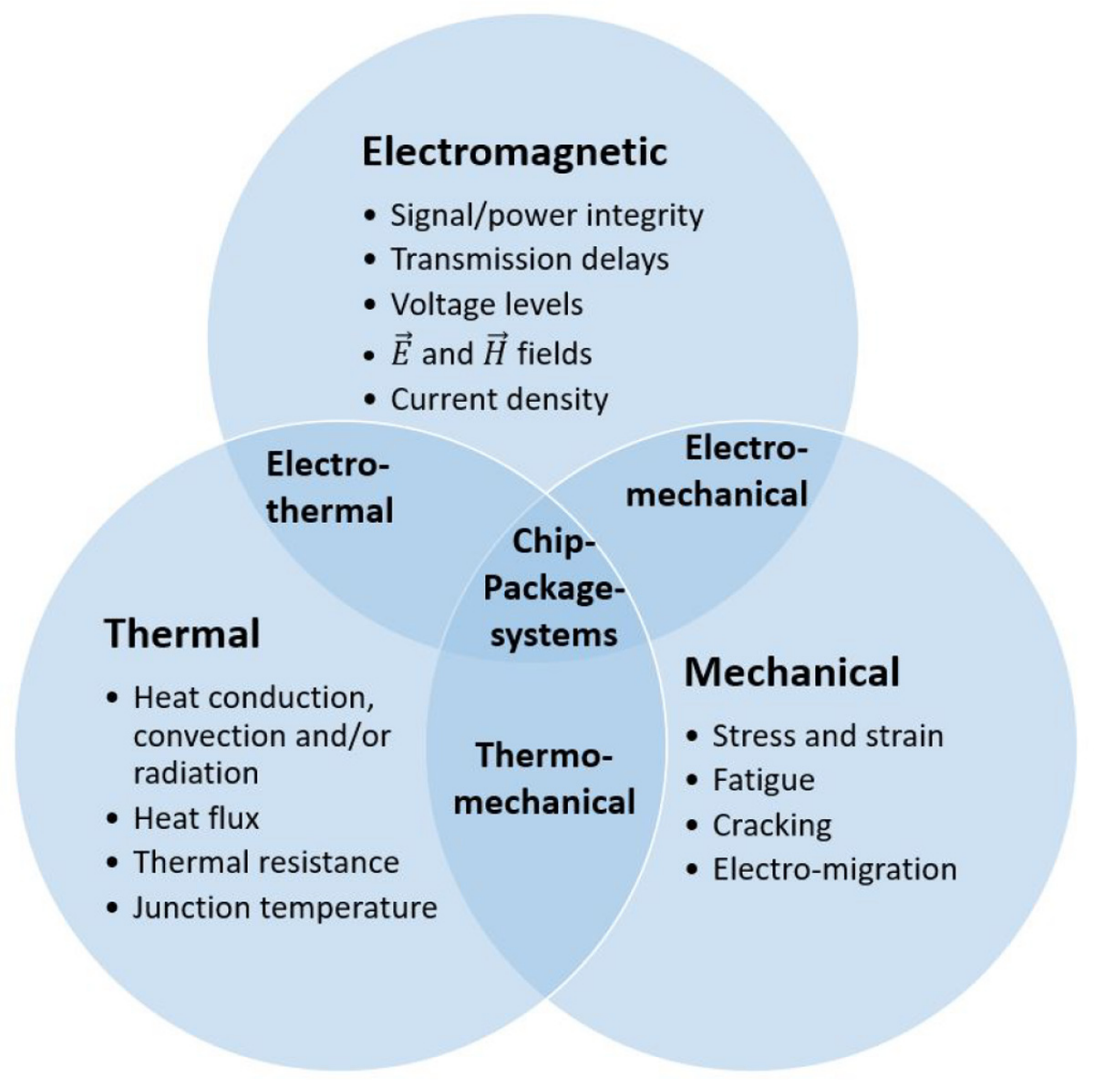
A diagram showing interactions between the physical domains.

Figure 7 represents the key physical phenomena that show how the electrical, thermal and mechanical domains interact with each other, demonstrating the need to co-design across chip-package-system domains and accurately model interactions ^59^. For example, power distribution at the electro-thermal interface should map accurately from a chip model into a package and system model. At the thermo-mechanical interface the accurate prediction of stress in the regions of the through silicon vias (TSV’s) is necessary as it impacts the transistor threshold voltages and drive currents. Similarly, at the electro-mechanical interfaces accurate prediction of stresses helps in modelling metal migration at the chip level.

Multi-physics simulation methodology, can play a vital role in 5G and beyond in developing a hardware architecture for antenna and base station, by predicting better design decisions and rapidly prototyping and optimizing these designs before fabrications which results in low cost, high performance, reliable and scalable hardware architectures.

***3.6.3 Multi-scale and multi-technology EM simulations*.** Going towards 5G and beyond, the trend in mmWave design advances towards smaller, more complex devices and higher frequencies. System in Package (SiP), System on Chip (SoC), Antenna in Package (AiP), Antenna on Chip (AoC), 3D die stacking ^60, 61^, just to name a few, all imply a larger number of closely placed devices. In these complex, scaled down designs, it is becoming increasingly difficult to electromagnetically isolate all the design components. This trend increases the importance of an accurate EMI/EMC behavior prediction from which stems the need for all-encompassing and accurate EM simulations ^62^. The increasing complexity and cost leave little space for iterative design changes after the device has been manufactured, which makes the EM simulation a significant element of the design process.

The most widely used simulation technologies are the finite element method (FEM), the finite difference time domain (FDTD) and the method of moments (MoM). Choosing the most adequate method to simulate a design with depends on the structure of the design itself. MoM being more suitable for planar designs, FEM for more complex 3D structures and FDTD for designs where the transient response is an important factor ^63^.

All these techniques imply the discretization (meshing) of the design domains prior to solving the Maxwell equations to calculate the EM fields for a particular design. An all-encompassing EM simulation on a common modern mmWave electronic system would require discretization of the technologies spanning over dimensions ranging from 10 ^−8^m on the IC level all the way to 10 ^−1^m on the printed circuit board (PCB) level. The goal of a good discretization method is to find a good trade-off between accuracy and computational resource usage efficiency. Preserving a good accuracy - resource usage trade-off throughout the whole design, poses a great challenge for modern EM simulation tools.

Regardless of what simulation technology is used, the simulation of these complex multi-scale designs can frequently become a computationally intense problem, solving of which requires more memory and time than available. Another approach to tackle this problem is based on the *divide-and-concur* strategy – the domain decomposition method (DDM) and it implies splitting the design into sub-domains and simulating those sub-domains with a simulation technology the most suitable for that particular design structure. This approach can either be mathematically inspired – solving large matrix systems by splitting them, or design inspired – the split at the hierarchical design levels. Although the DDM methodology has been shown to improve the efficiency of the EM simulations in some specific problems, such as those described in 64 and 65, the study of the generalization of this principle is not yet available.

## 4 MmWave antennas

One of the most desired frequencies for 5G due to its spectrum availability is 28 GHz. Successful antenna designs with 1 Gbps throughput and high output power are demonstrated in phased-array architectures ^66, 67^ and in massive-MIMO with hundreds of elements ^68, 69^. It is envisioned that the 5G base stations would use up to a thousand antenna elements ^70^.

The next goal for 6G – or what is often called beyond-5G – would be to achieve 1 Tbps data rates ^71^. For that, the operating frequencies of antennas must increase even further, to above 100 GHz. Higher frequency means less range due-to over-the-air path loss ^72^, higher transmission line losses ^73^ and more interconnection losses ^74^. Another issue is rapid decrease of efficiency of the power amplifying ICs with frequency ^75^, to a point where the state-of-the-art PAs at 140 GHz have the power added efficiency of 6-7 % ^76^. Section 5 discusses these issues in more details, e.g. see Table 5 for references. All of these mean that most of the power generated at 100+ GHz is dissipated in the system as heat. Thus, it is not enough just to scale down the current transceiver chips proportional to the wavelength since we will end up with prohibitively high power consumption. Therefore, new design approaches are required for sub-THz frequency ranges.

### 4.1 Antenna element design and array scaling

The choice of antenna element topology is a starting point of antenna system design. It involves a variety of considerations: the substrate technologies, the target performance (bandwidth, gain, radiation pattern), the required inter-element spacing (usually half wavelength) that defines the array size, and interconnection flexibility to allow radio-frequency integrated circuit (RFIC) assembly and multi-board integration in a system.

Phased array approach has a long history and is still in trend for state-of-art antenna development. The effective isotropic radiated power (EIRP) of an N-element phased array grows as *N* ^2^ while the power consumption grows linearly with *N*, which provides a very efficient way to generate power in space. In 5G era, automotive radar, backhaul communications all require multiple point-to-point data links simultaneously, for which large phased arrays that produce multiple steered beams in various directions are favored. These beam-forming functions are realized by phase shifting among the antenna elements, which can be implemented in analogue circuits operating at RF, local oscillator (LO) or intermediate (IF) frequency, or in digital circuits ^77^. Therefore, it is important to consider integration or interconnections design between the beamforming IC and the antenna elements/subarrays. As the frequency goes up to W-/D-band, the size of integrated active IC becomes comparable to the antenna element, which makes it harder to cascade independent beamformer to each element in phased array, thus posing a challenge to keep a large number of elements (higher radiated power) and tight element spacing (large angle beam steering) simultaneously. The general strategy for creating large phased arrays by combining repeatable circuit and/or antenna units is called phased-array scaling ^78^.

At lower frequencies, many microwave systems consist of separate modular ICs and PCBs connected to each other on a common carrier board. The RF signal at these frequencies can be routed with tolerable losses between different chips via the transmission lines on the carrier board. This type of horizontal integration is also referred to as multichip modules (MCM). This approach is great for applications that prefer simplified integration and parallel component development, but at mmWave frequencies the interconnection losses become prohibitively high.

For better RF performance and further miniaturization, it is desirable to bring the sub-system components closer to each other. One solution is to package the analog, the digital and the antenna chips in the same packaging process step, creating an Antenna-in-Package (AiP). The main challenges here are the system complexity and heat dissipation ^79^.

Another solution, with a higher level of integration, is to fabricate the sub-system elements on a single die. In this approach, the antenna is matched directly to the IC on the same chip. This Antenna-on-Chip (AoC) concept is especially promising for 100+ GHz frequencies where the size of the radiating elements become small and it becomes feasible to allocate the expensive chip area for antennas. The main drawback of this approach is that the ICs are often fabricated on high-permittivity and low-resistivity silicon substrates. The former means that a lot of radiation is pulled into the substrate, and the latter makes this radiation dissipate in the substrate as heat. Another problem is spacing: it might not always be possible to fit the PAs,LNas and other components between the antenna elements while simultaneously satisfying the *λ/*2 inter-element separation and providing input/output signal routing.

### 4.2 Phased array packaging solutions

**Figure 8.**
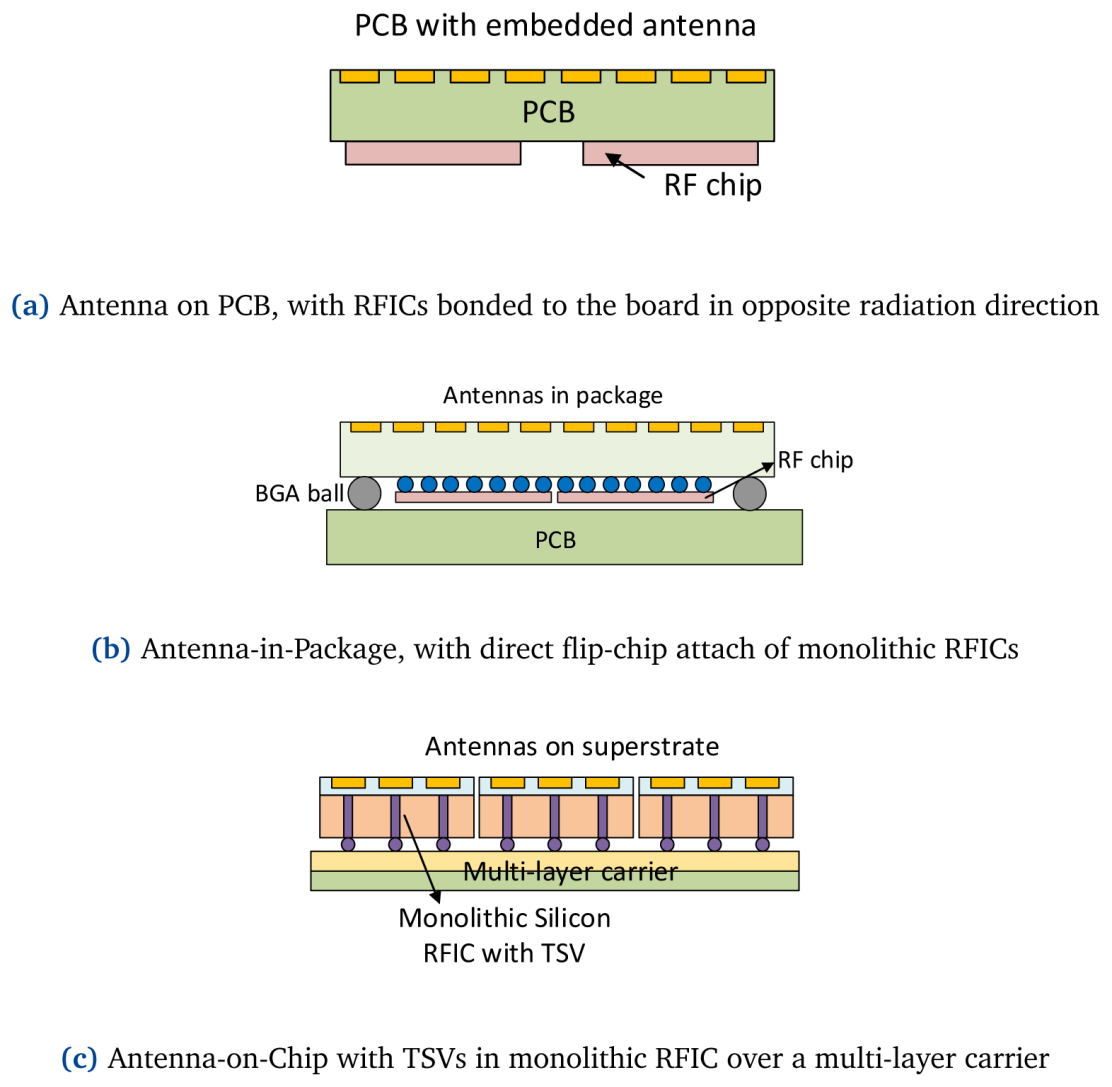
Classification of system packaging implementation diagram with different approaches. RF, Radio Frequency; BGA, Ball-Grid Arrays; PCB, Printed Circuit Board; RFIC, Radio-Frequency Integrated Circuit; TSV, Through Silicon Via.

The low-cost Antenna-on-PCB solution is widely used in lower mmW designs. As Figure 8(a) shows, antenna arrays are implemented directly on the application PCB, with the RFICs module bonded to the board on the backward-radiating direction. PCB process allow flexible choices for printed antenna types that offer wide-band or wide-scan performances. However, due to PCB manufacturing tolerances when the frequency shifts higher and antenna scales tighter, it is difficult to combine on-chip RFICs with large numbers of array elements, which restrains the technology to be applied at 60+GHz bands. In 2018–2019 authors of Nokia Bell Labs presented a tiling architecture with one IC per 24-antenna tiles in a 384-element phased array for point-to-multipoint applications ^80, 81^. Two organic PCB interposers with integrated antenna sub-arrays were designed and co-assembled with the RFIC chipsets to produce a scalable phased-array tiles. The tiles were aligned onto a carrier PCB to form the large phased array. By far it’s the most elements, most signal chains and highest EIRP of reported W-band AiP solutions.

To reduce the interconnections between discrete antennas and beamformer ICs, Antenna-in-Pacakge (AiP) technology implements antennas with transceiver dies into a standard surface-mounted device ^79, 82^. AiP approach implements the antennas on the 1st-level package, see Figure 8(b). The antenna array with filp-chip bonded ICs form the 1st module, which is attached to a 2nd PCB board connected through ball-grid arrays (BGAs). Its main advantage is that the 1st-level embedded subarrays can be duplicated to form large arrays. In 2014, authors from IBM presented a 94 GHz four-IC phased-array tile with 64 dual-polarized antennas embedded in a multilayer substrate, with four SiGe (silicon-germanium) transceiver ICs flip-chip attached to the package by 292 BGAs ^83^.

### 4.3 On-chip antennas

**Figure 9.**
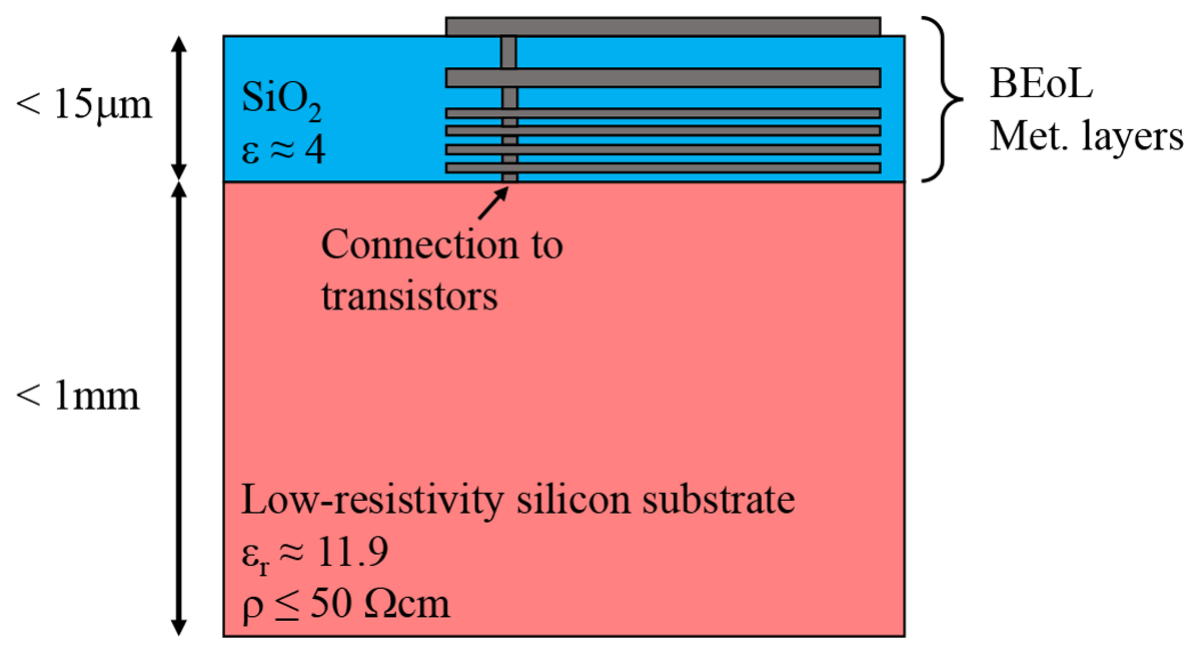
A schematic view of a typical silicon-germanium (SiGe chip). BEoL, Back-End-of-Line.

The third packaging approach is to design antennas directly on the RFIC wafer, which eliminates the transition loss from the chip to the antenna by using high-efficiency on-chip quartz superstrate [see Figure 8(c)]. A typical IC consists of a thick high-permittivity substrate (Si, GaAs, GaN etc.), with an active region on top of it which is referred to as Front-End-of-Line (FEoL). Transistors and other active circuitry are formed on the FEoL layer. A thin dioxide layer, known as Back-End-of-Line (BEoL), which incorporates several planar metal layers, is grown on top of the FEoL. The BEoL is used to carry the signals to and from the FEoL, and to connect the IC to other devices. A typical SiGe chip (not to scale) with its different layers is depicted in Figure 9.

**Figure 10.**
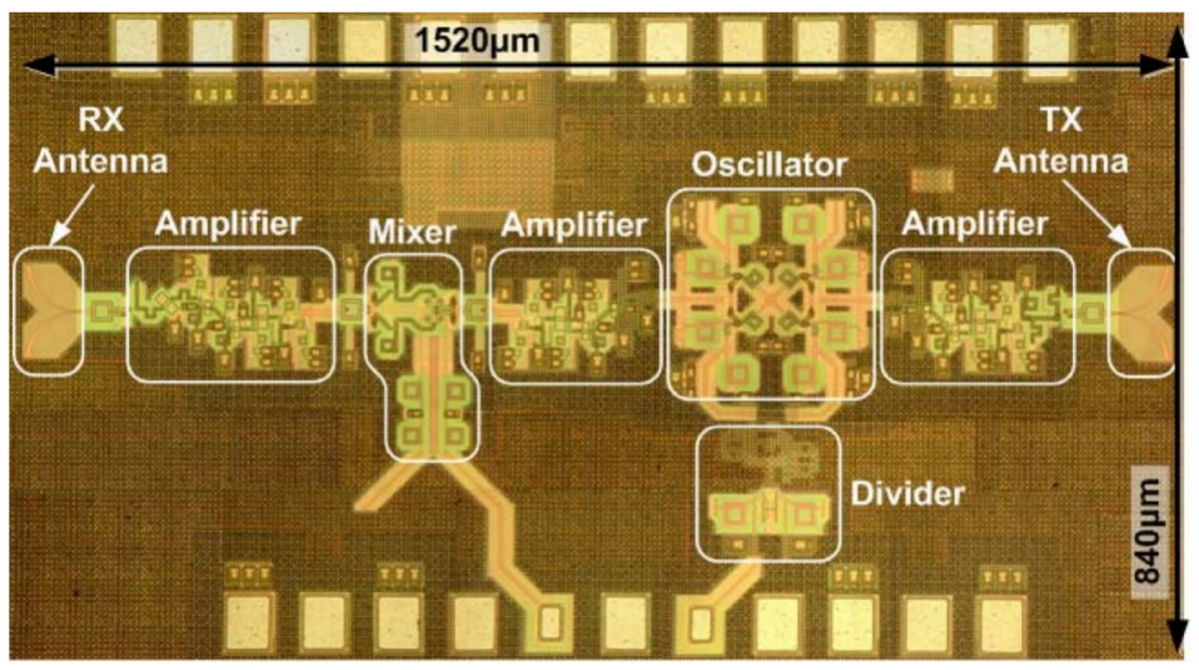
An example of a silicon-germanium (SiGe) antenna-on-chip. RX, Receiver; TX, Transmitter. ©2008 IEEE. Reprinted with permission, from 92.

Antenna-on-chip (AoC) is often formed in the top metal layers of the chip’s BEoL. By fabricating it on a chip we minimize the distance between the antenna and the active region where the signal amplification is happening (somewhere under Metal 1 in Figure 9), which reduces the interconection losses and allows for better integration when compared to AiP approach. Modern advances in SiGe BiCMOS technologies promise excellent RF performance up until THz range ^84^. A whole transceiver chain at 100+ GHz can be fabricated in this technology occupying less than 1.5 mm ^2^ chip area ( Figure 10).

It is, however, very challenging to achieve good radiation performance of an antenna fabricated on a silicon substrate. Most of the antenna radiation is pulled into the substrate due to its high permittivity. This then couples to the substrate waves that dissipate as heat or re-radiate in undesired directions ^85, 86^. One solution to this issue is to ground off the substrate by placing the ground plane inside the BEoL, which in terms of Figure 9 means to use the bottom metal layer (for example) as a ground. This way we remove the silicon substrate from the equation, but the antenna now is placed too close to the ground plane, which reduces its bandwidth and radiation efficiency ^87^. Another solution is to use cavity-backed antenna designs ^88^, which may improve the radiation efficiency. However, it does not solve the problem completely and the device performance will still heavily depend on the chip geometry. When one designs an antenna-on-chip array, a smart placing of the antenna elements can result in substrate waves attenuation ^89^, but this approach puts a lot of constraints on practical array implementations. With recent successes in miniaturization and development of advanced packaging techniques, it is becoming very popular to use metamaterials that can attenuate the undesired waves propagation in the system ^90^. Metamaterials can also be used, for example, to tune the substrate wave dispersion relation in a controlled way which allows drastic array radiation efficiency improvements ^91^.

## 5 mmWave electronics SoA

The broad-spectrum bandwidth and complex modulation techniques adopted in the 5G wireless communication systems pose significant challenges to the design of millimeter-wave (mmWave) front-ends. In addition to the stringent specifications imposed by the operational requirements of 5G wireless communication systems, the high frequency of operation makes the design of mmWave front-ends circuits such as low noise amplifiers (LNAs), phase-locked loops (PLLs), phase-shifters (PSs), mixers, and power amplifiers (PAs) more challenging than at lower frequencies. This is due to the power losses, the sensitivity to components tolerance, and the physical limitations of the transistor technologies. This section reviews state-of-art (SoA) mmWave front-end circuits, specifically LNAs, PLLs, PSs, mixers, and PAs.

### 5.1 Radio frequency front-end non-idealities

**Figure 11.**
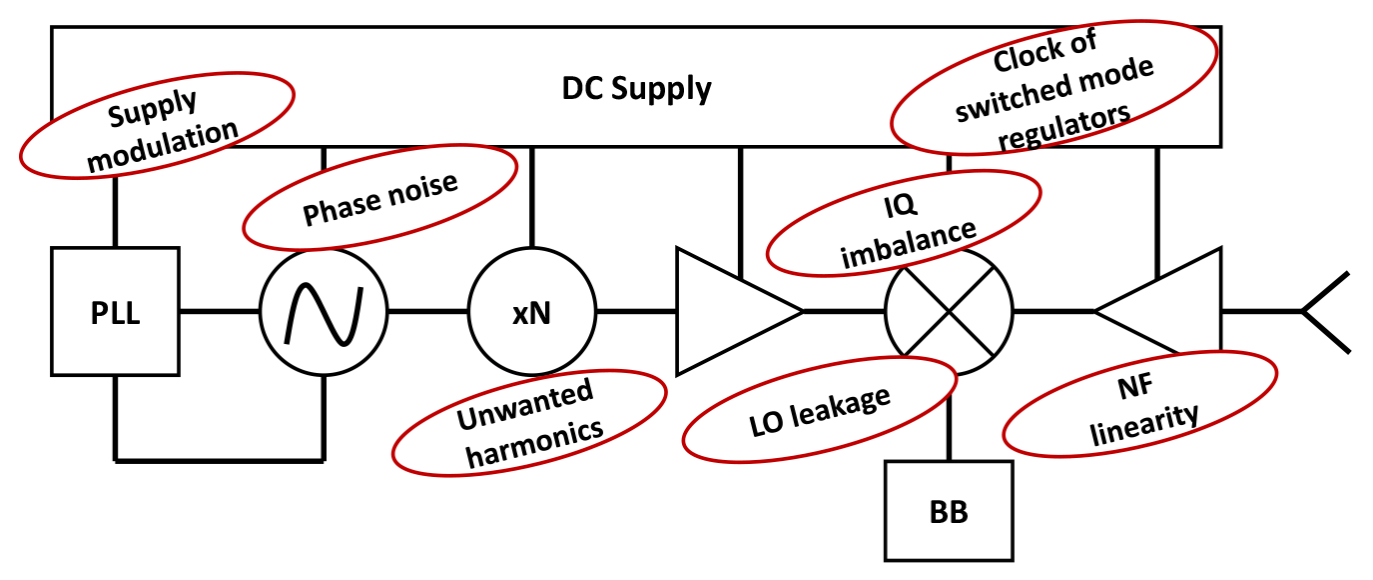
Non-idealities of the Radio Frequency Front-Ends (RFFE). PLL, Phase-Locked Loops; IQ, Inphase-Quadrature; LO, Local Oscillator; NF, Noise Figure; BB, Baseband.

Through the advancement of the mobile communication technologies, each generation lead to higher effort on the design of the base station (BS) or AP and UE radio frequency front-ends (RFFEs). Both are, in principle, implemented with the same building blocks, such as amplifiers, filters, antennas, mixers, etc. Consequently, they are prone to have a reduced performance due to the components non-idealities. There are many unwanted signals generated in the RFFEs as depicted in the Figure 11, where even the power supply imperfections would result in a signal modulation.

In the local oscillator (LO), the phase noise is an undesired property that is usually kept as low as possible by design to obtain a higher spectral purity. The quality factor of resonators in oscillators degrades with frequency. Therefore, to reduce the phase noise and achieve fair quality factor, frequency multipliers are implemented between a lower frequency LO and the mixer. With this configuration the module is often called sub-harmonically pumped mixer, as it uses harmonics of the LO to mix (or up/down convert) signals, this practice also contributed to increase the mixer port isolation in around 20-30 dB ^93^.

**Figure 12.**
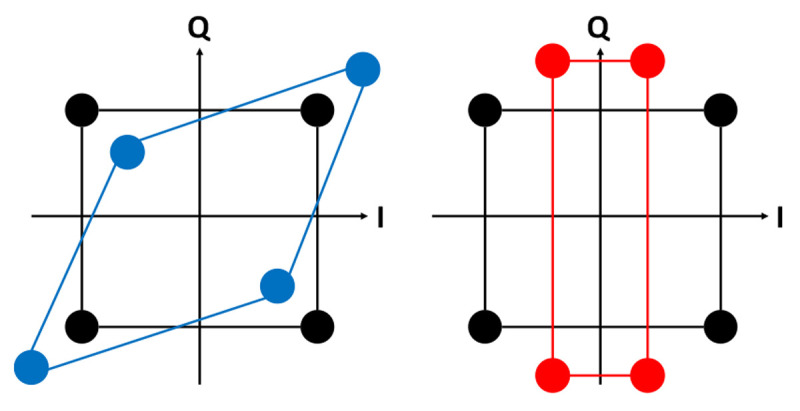
Inphase-Quadrature (IQ) phase (left) and amplitude (right) imbalance.

The inphase-quadrature (IQ) amplitude and phase imbalance ( Figure 12), affects the signal (symbol) detection and methods to overcome this effect have been developed. In the physical design of mixers, it is known that the LO port must be driven into saturation as it compensates the non-uniform baseband signal strength, so the amplitude impairment of the baseband signals are not a major problem, but as the LO port manages a high power, the isolation should be considered for circuit design.

The carrier frequency can be generated with a voltage controlled oscillator (VCO) attached to a PLL circuitry to stabilize the LO and in many cases using frequency multipliers. The frequency multipliers and mixers generate unwanted harmonics and intermodulated products that may propagate towards the PA, increasing the adjacent channel power ratio.

**Figure 13.**
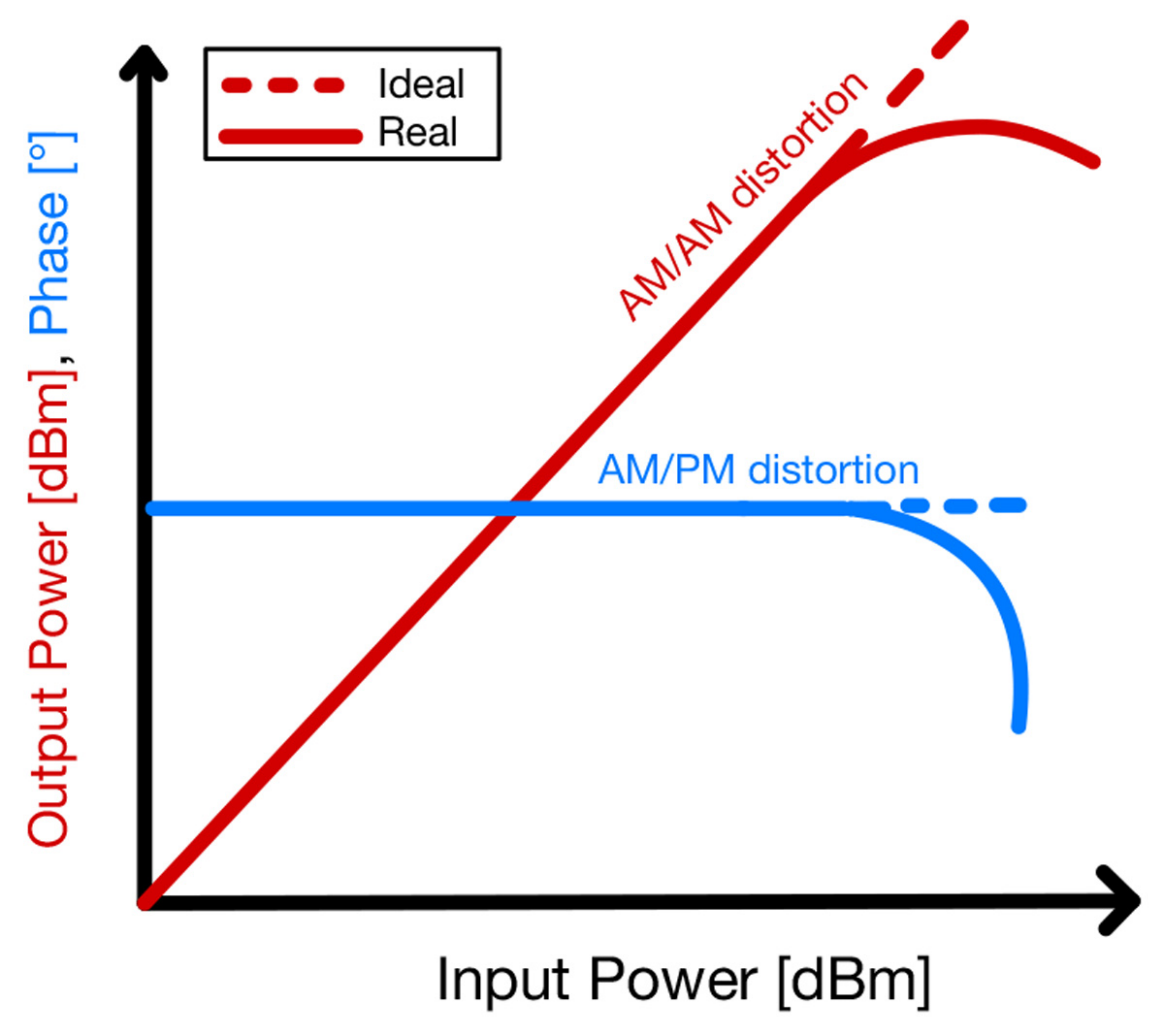
Amplitude and phase distortion in a Power Amplifier (PA). AM/AM, Amplitude-to-Amplitude Modulation; AM/PM, Amplitude-to-Phase Modulation.

The amplifiers have different requirements if they are implemented in the receiver (RX) or the transmitter (TX) path. The amplifiers for the RX path are called LNA providing low NF at the cost of lower power handling capabilities. The NF is an important factor from the transceiver chain to consider in the link budget, where all the power gains and losses in the signal path are accounted. On the other hand, for the TX path, PAs are used, as they provide a larger output power which is routed to the antenna for the propagation of the signal. The PA is probably the most non-linear element and this may handle the largest power in the whole TX. The non-linearities of a PA can be observed in amplitude-to-amplitude modulation (AM/AM) and amplitude-to-phase modulation (AM/PM) as shown in Figure 13, distorting the gain and output phase of the amplified signal, respectively. The more saturated is the PA, the higher is the distortion. These effects can be reduced with pre-distortion techniques. Furthermore, the PA should operate efficiently, because a high temperature of the transistor channel, reduces its median life time before failure.

### 5.2 Local oscillator distribution techniques in MIMO systems

Achieving synchronization in terms of frequency and phase between several of the tiles (where each of the tiles host a number of on-chip transceivers and local oscillator architecture usually implemented as phase locked loop) on a base station poses a critical performance bottleneck; if left unattended it can prove to have disastrous affects for phased array performance of network ^81, 94, 95^. To make several channels of transceivers phase coherent from a base station therefore is of paramount importance. The approach usually adopted is to make use of some calibration mechanism that tends to remove phase offsets between the transceivers of each channel periodically to make them more coherent.

These phase offsets exist within the transmitter, receiver architecture as well as within local oscillator architecture.

However, the synchronisation process for local oscillators is complicated by the fact that it involves two fundamental problems of phase noise and LO drift as function of temperature & time. Both issues are random with respect to time and cannot be calibrated out. In such an instance two obvious choices would be to either

•  Reduce phase noise and LO drift to such an extent that channel-to-channel variation does not remain a bottleneck. 

    Or

•  To design a LO sharing architecture such that channels become coherent despite the variations.

One of the most popular techniques currently being employed in state of the art of Massive MIMO systems is the H-tree technique ^96^. In this method, a single Reference Clock is routed to each of the phase locked loops in each tile making use of power divider ensuring synchronised behaviour. One of the latest works consisting of all digital PLLs has incorporated this technique for achieving synchronisation in a phased array. In this work ^96^ phase array performance is demonstrated at 60 GHz using digital beam steering capability for MIMO transmitter by making multiple all digital Phase locked loops (ADPLLs) phase coherent using calibration method. Although having easy design, this method suffers from some critical limitations; namely scalability issue, power constraint on reference clock to drive many loads, requiring external hardware & software calibration mechanism to calibrate out the phase offsets.

**Figure 14.**
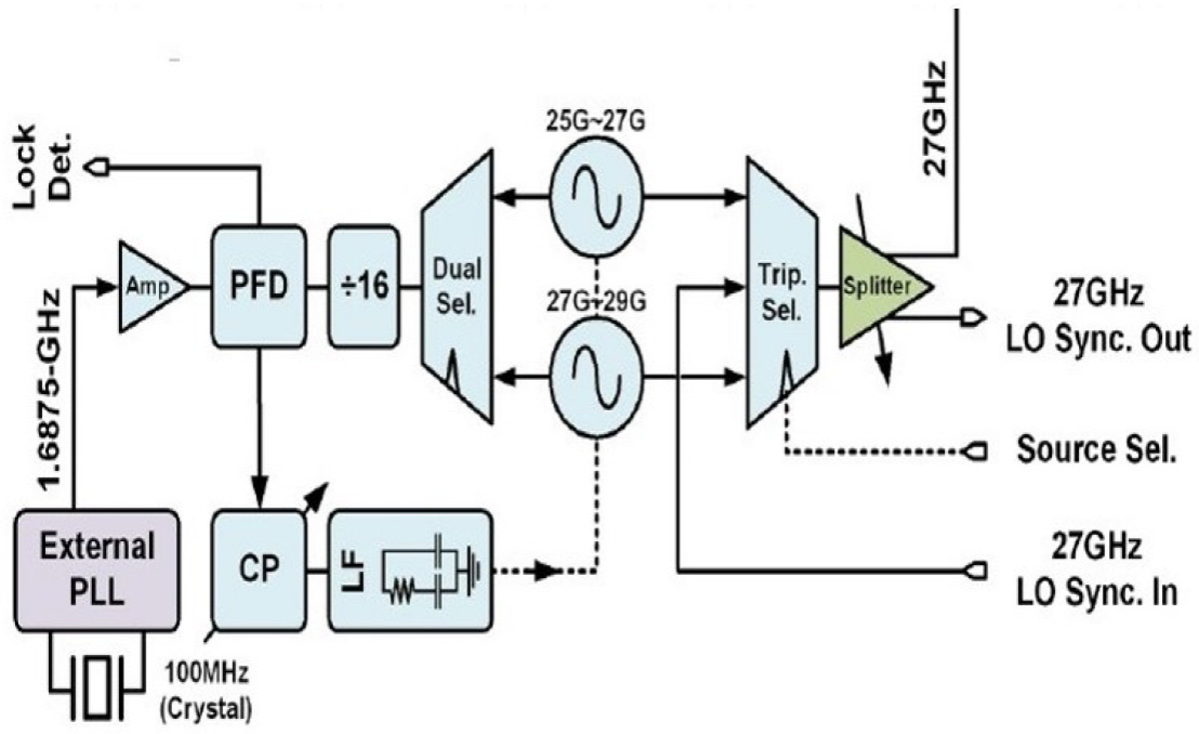
Daisy chain technique. PLL, Phase-Locked Loops; LO, Local Oscillator; CP, Charge Pump; LF, Low Pass Filter; PFD, Phase Frequency Detector. ©2019 IEEE. Reprinted, with permission, from 81.

Another technique that has been used lately is termed as daisy chain technique ^81^ ( Figure 14). Its principle of operation is that each phase locked loop becomes a reference clock for each subsequent phase locked loop. Reference clock itself only feeds the first phase locked loop. Complex distribution network for reference clock is no longer needed (reduced footprint) and no constraints on the output power of reference clock. This technique has been intelligently adopted recently ^81^ by demonstrating a W-Band integrated 384 element phased array. In this work instead of using the output of each PLL directly as input, a frequency lower than output frequency (90GHz) at 27 GHz clock is used between the slave nodes. This reduces incoherence between channels due to LO drift because of lower multiplication factor and lower amount of voltage noise being translated to time noise as a result.

Daisy chain technique has a limitation however, as the number of PLL increases, the phase noise progressively becomes poorer in performance resulting in more incoherency among channels.

**Figure 15.**
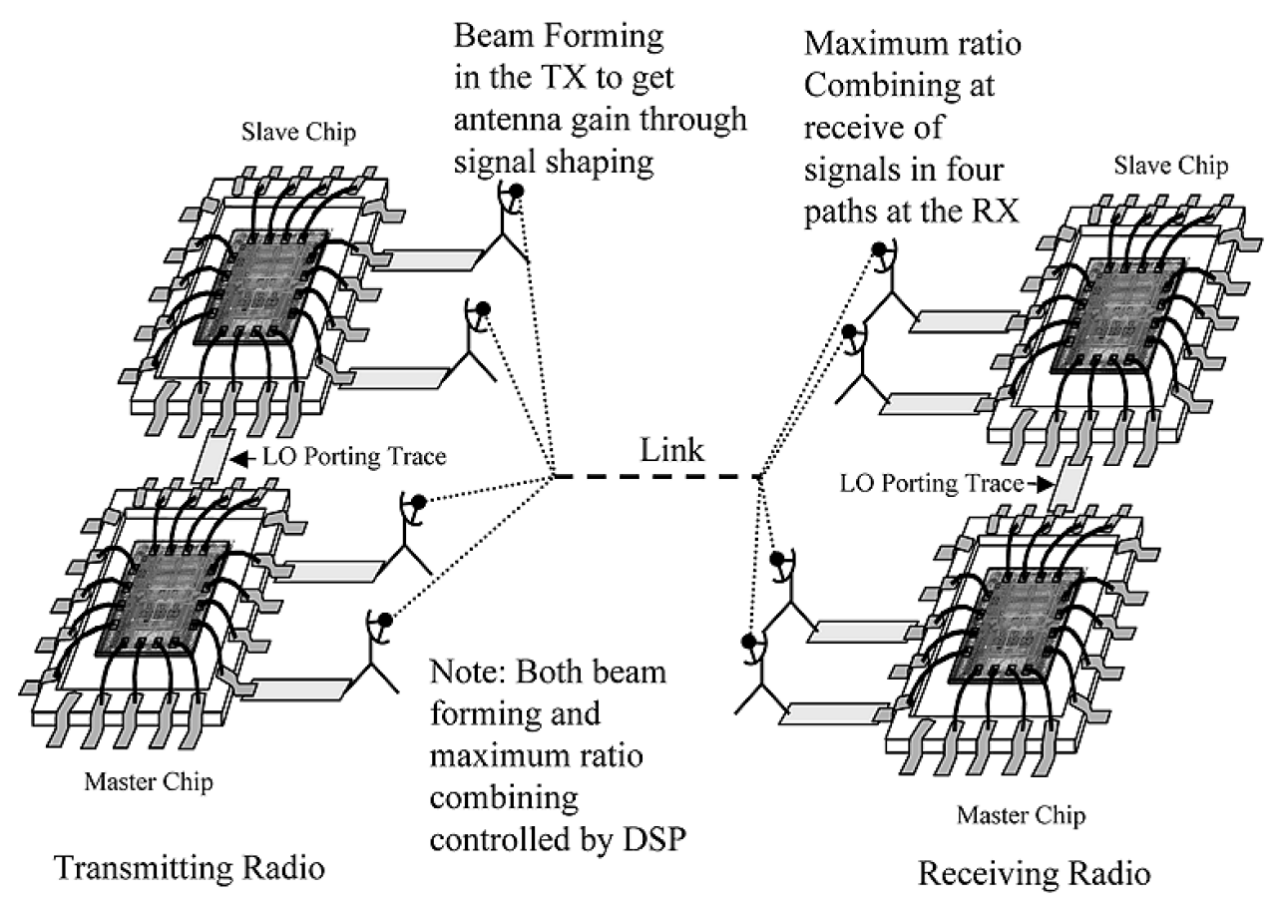
Star coupled technique. RX, Receiver; TX, Transmitter; DSP, Digital Signal Processing; LO, Local Oscillator. ©2005 IEEE. Reprinted, with permission, from 94.

Another technique which is closely related to daisy chain method is called star coupled ^94^ technique. In this architecture, an optional LO porting bock is added such tat it allows of sharing of a common system RF-LO among various chips. With this mode, the LO from one Maser chip is distributed to all the slave chips as shown in Figure 15 driving their clock trees and dividers. Despite some fixed delay associated with LO distribution, many drifts related mechanism are dealt with.

**Figure 16.**
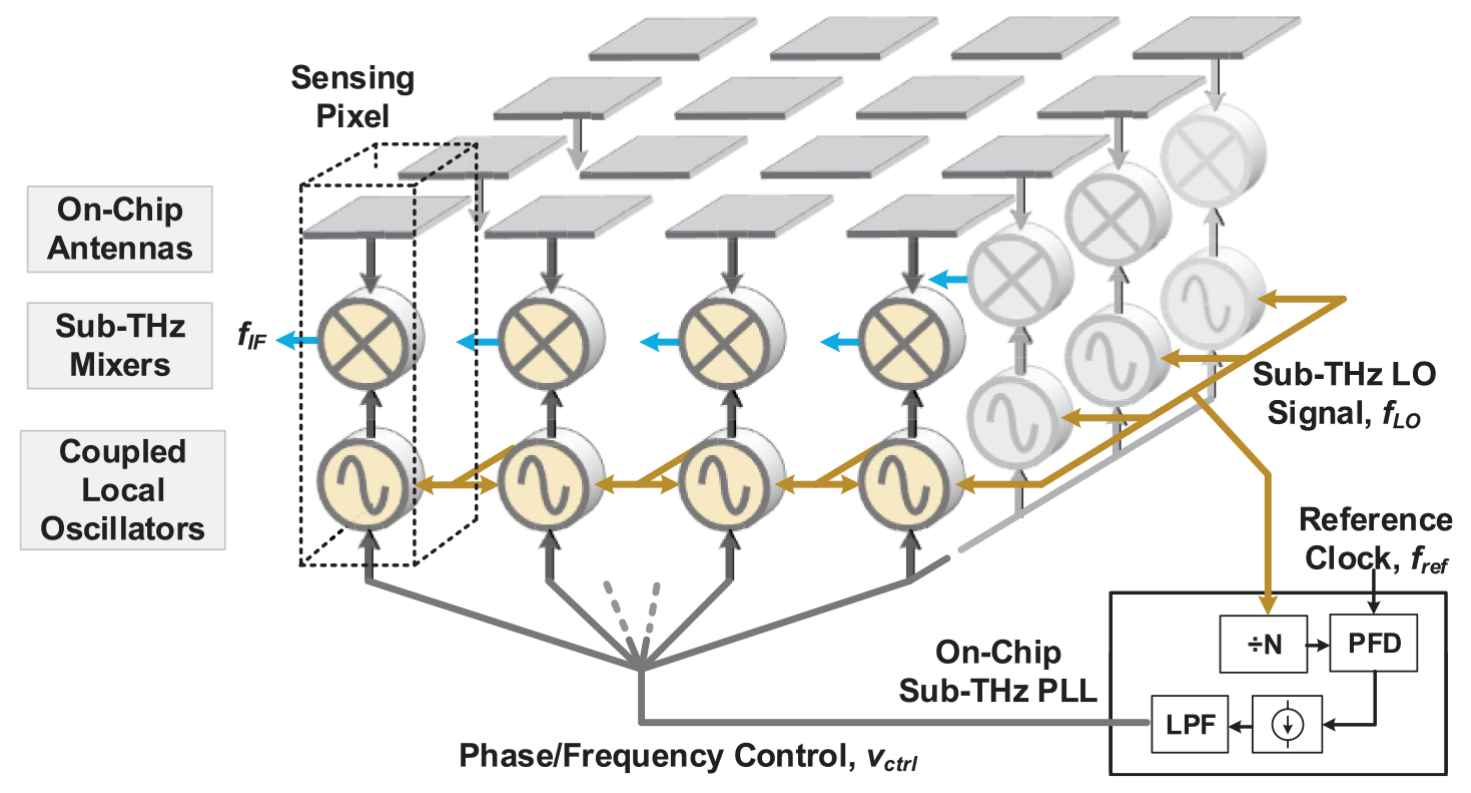
Coupled oscillators technique. LO, Local Oscillator; PLL, Phase-Locked Loops; PFD, Phase Frequency Detector; LPF, Low-Pass Filter. ©2019 IEEE. Reprinted, with permission, from 95.

The techniques of coupled oscillators ( Figure 16) and coupled phase locked loops have recently emerged as superior alternatives. The techniques were originally employed as alternatives to analog phase shifters by using oscillator elements directly to create the required phase offsets making use of injection locked technique.

The work ^97^ for example is based on coupled oscillator theory to generate multiple phases, Both the simulation and experimental results of the work show that the as the Number of oscillator cores N increases, the phase noise decrease by the amount 10log10N , and the number of phases required can be expanded easily by adding one more resonator core.

Similarly, in 98 it’s shown how phase controlling signals are applied separately to each of the oscillators in order to de-tune their natural frequencies of each of the oscillators, while a common injection locked signal is provided to each of the oscillators via power distribution network. This method not only aligns the oscillators to common frequency of injection locked signal but also allows control of constant phase progression as one move across the linear array of elements. Thus, it allows an efficient method of beam-steering the phase array without incurring additional power & area employed usually for analog phase shifters. This methodology is especially very useful for mmWave based phased arrays where substrate space is scarce. . Although the injection locked oscillators seem like a convenient solution to beamforming in phased arrays or to achieve coherent output signals, they have some limitations inherent in their architecture. The phase dynamics and locking range of Injection locked oscillator depends on the quality factor of oscillator, the magnitude of the injected signal strength. Thus, they suffer from low locking range, and non-uniform amplitude of output waveform in the instance of frequency detuning, leading to more serious problems once the array size increases. Thus, coupled PLL technique ^99– 101^ offers superior results as far as locking range and amplitude performance is concerned. The type of PLL architecture also affects the overall performance metrics of coupled phase locked loop system. Circuit fabrication inconsistencies lead to changes in the free-running frequencies of oscillator which as a result cause phase errors and affect the beam-pointing performance. Likewise, these fluctuations can also impact the common frequency of oscillator array leading to deviation from the design frequency. These fluctuations also prove to be a bottleneck in the performance of coupled Phase locked loops if PLL is of Type-1 topology ^100^. With the type-2 PLL topology adopted, the performance proves to be much more stable in terms of output frequency and the resilience of architecture against such fluctuations.

**Figure 17.**
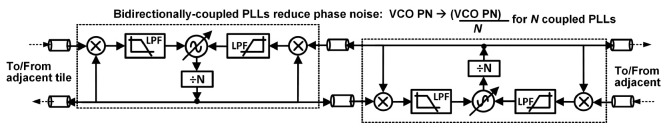
Coupled phase locked loop technique. PLL, Phase-Locked Loops; LPF, Low-Pass Filter; VCO, Voltage-Controlled Oscillator; PN, Phase Noise. ©2016 IEEE. Reprinted, with permission, from 101.

The work in 101 shown in Figure 17 is the most recent and promising result as far as application of coupled PLL in the synchronisation is concerned. The work improves upon the limitations of two-wire bidirectional coupling of phase locked loops by making use of single wire architecture using quadrature coupler at the input of each of the phase detectors in a single PLL IC. The architecture reduces the phase noise and is also a compact solution compared to two wire approach and can be readily employed in the tiled approach scaling of MIMO systems. Additionally, the structure make use of Type-2 PLL and includes variable phase shifters in each PLL cell to compensate for static phase offsets between the LO ICs which is undesirable for an application that doesn’t make use of phase array performance into its design.

### 5.3 mmWave based oscillator

The continuous push to realize integrated digital intensive radar chip transceivers and wireless communication systems operating at mm waves has paved way for CMOS to become the natural technology of choice. Since the latest nanoscale technologies offer transit frequency (ft) and maximum oscillation frequency (fmax) above several hundreds of gigahertz ^102, 103^. While the voltage control oscillator (VCO) lies at the heart of any transceiver system, it simultaneously must fulfill a set of conflicting requirements including low phase noise, wide tuning range, and high operating frequency. The lowest phase noise is required to enable higher modulation schemes in high data rate communication systems or to achieve better target discrimination in radar systems. While wide tuning range is essential for high resolution required in continuous wave radar sensor in gesture recognition or automotive. This necessitates the use of large varactors that owing to low quality factor exhibit poor phase noise at mm waves ^104^.

Voltage controlled oscillators can be categorized into two flavors: RC ring based, and LC based. The ring-based oscillators consist of several delay cells such that Barkhausen criteria is met. Advantage of this topology is absence of integrated inductors leading to more compact and wide tunable solutions; but suffers from a significant higher phase noise therefore is not often used in mm wave-based design. The highest mm wave frequency to have reported a ring oscillator is at 32 GHz ^105^. The LC oscillator consists of a resonant tank, the losses of which are compensated by a negative resistance provided by active circuitry. Finally, there are also options for distributed oscillators to be implemented in CMOS ^106^ but at the expense of higher design complexity and larger chip area. The distributed standing wave topology is more favored toward 300GHz frequency ^107^.

Designing and running a VCO at mm wave frequency poses additional constraints compared to classical VCO designs at few gigahertz of RF. The quality factor of switched capacitors and varactors fall rapidly with frequency leading to phase noise degradation as well as small output power ^108^. Commonly this issue is alleviated by designing a VCO at lower mm wave frequency in conjunction with frequency multiplier ^109^, extraction of harmonics ^104, 110, 111^, and subharmonic injection locking technique ^112^. The flicker noise corner also progressively becomes poor with the downscaling of CMOS technology node as well as the flicker noise contribution becomes worse at mm wave frequency Another major bottleneck is imposed by device parisitics and passive interconnects. These parisitics capacitances could add up to wanted capacitance and degrade the tuning bandwidth ^108^ and become especially challenging with reconfigurable passive resonators ^113^ as well as VCOs employing capacitive divider ladder networks ^114^. Furthermore, the coupling between the neighboring inductor coils increases greatly at mm wave frequencies and needs careful modeling and shielding techniques ^115^. Finally, the parasitic inductance of traces could become comparable the actual inductance because of reduced size of inductor at these frequencies and negatively impacts the performance of system ^108^.

There is a limit to which phase noise could be reduced with the proper selection and sizing of active and passive components. There are a couple of techniques that could be leveraged to reduce phase noise further. One of them is bilateral coupling of identical cores resulting in phase noise improvement of 10logN where N is number of coupling cores. The technique is bounded by practical limitations such as area and power constraints ^109, 116, 117^. Also, to extend the frequency tuning one can replace varactors by a transformer ^117, 118^. Similarly, the work ^119^ in proposed employing a loop ground transmission line to achieve high tuning range. Another similar technique that proves to be promising is inductive tuning ^120^. There is also another technique to extract higher harmonics from the same oscillator for example by realizing a push-push VCO one can extract second harmonic present at common mode of circuit by attaching a transformer ^104^. Alternatively, it’s possible to distinguish the second harmonic from fundamental by mode of signalling and second harmonic in common mode is capacitively coupled out ^121^. A third harmonic could also be extracted by making use of power amplifiers that boost the third harmonic while suppresses the fundamental and second harmonics ^110, 111^.

### 5.4 Mixers

While conventional solutions for mixers are remaining successfully implemented, circuit designers keep looking for possible improvements as well as integrating additional functions apart from solely up- and down-converting.

One of the possible enhancement techniques was demonstrated in 122. This work presents the 77 GHz down-converting mixer, based on the modified Gilbert cell. The main idea is to substitute the usual resistive loads with active ones, consisting of resistors and p-channel metal–oxide–semiconductor (PMOS) transistors. This solution is meant to stabilize the output DC voltage. In general, the presented mixer is power- and area-efficient, since P _*DC*_ of the mixer’s core is only 1.5 mW and the device occupies 0.065 mm ^2^.

**Figure 18.**
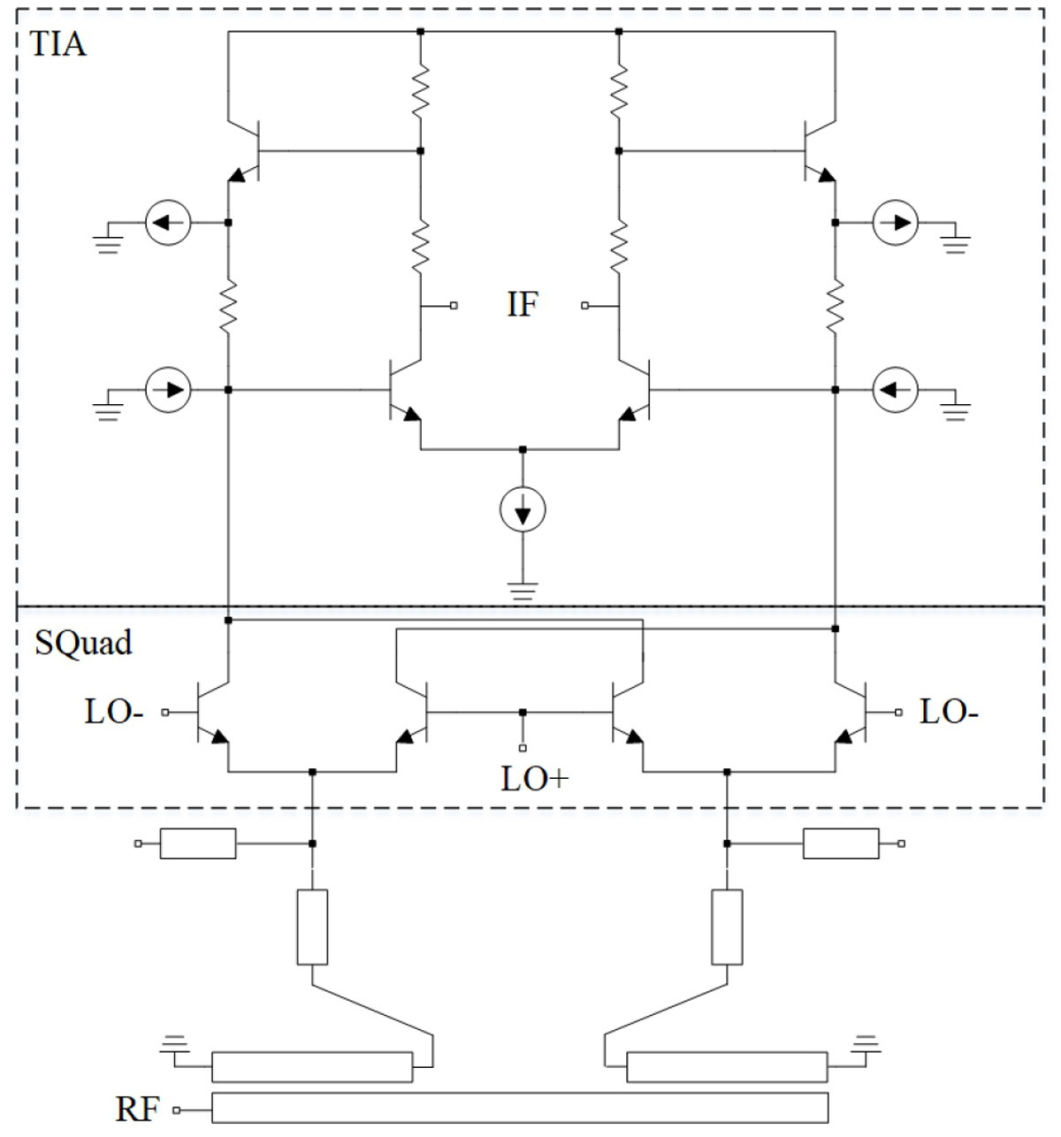
Mixer, loaded by a Transimpedance Amplifier (TIA). IF, Intermediate Frequency; LO, Local Oscillator; RF, Radio Frequency. ©2021 IEEE. Adapted from 123 with permission.

An interesting device integration is presented in 123 ( Figure 18). The authors used a transimpedance amplifier as a load for a broadband Gilbert cell mixer. Due to broadband RF matching network, RF signals from 110 GHz to 170 GHz can be down-converted with a conversion gain of higher than 20 dB.

**Figure 19.**
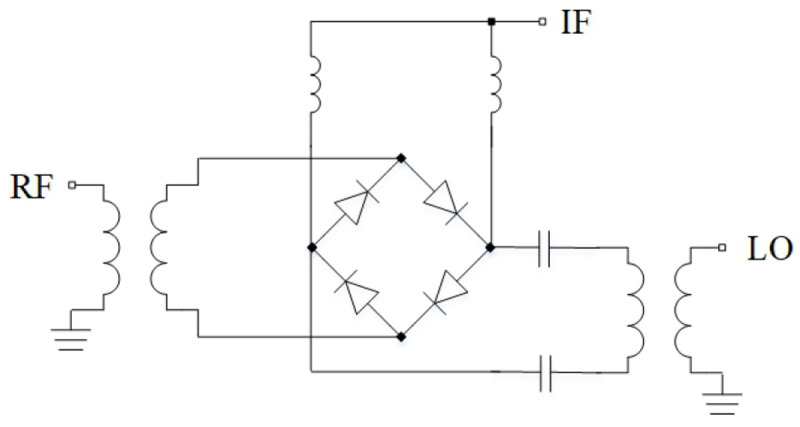
Mixer, based on diode ring. IF, Intermediate Frequency; RF, Radio Frequency; LO, Local Oscillator.

In general, both passive and active mixers have certain advantages. Passive mixers have zero power consumption and are highly linear and broadband devices. On the other hand, they require high LO power and exhibit high losses. Since passive mixers are still of interest, circuit designers try to make further improvements in such designs. Thus, presented in work ^124^ ( Figure 19) mixers are based on the conventional diode ring. Due to the use of wideband transformer BALUNs at the RF and LO inputs, the widest frequency range achieved was 5 – 50 GHz. The novelty of this work is the use of additional phase-shifting components in order to compensate various imbalances at the edges of the frequency range.

**Figure 20.**
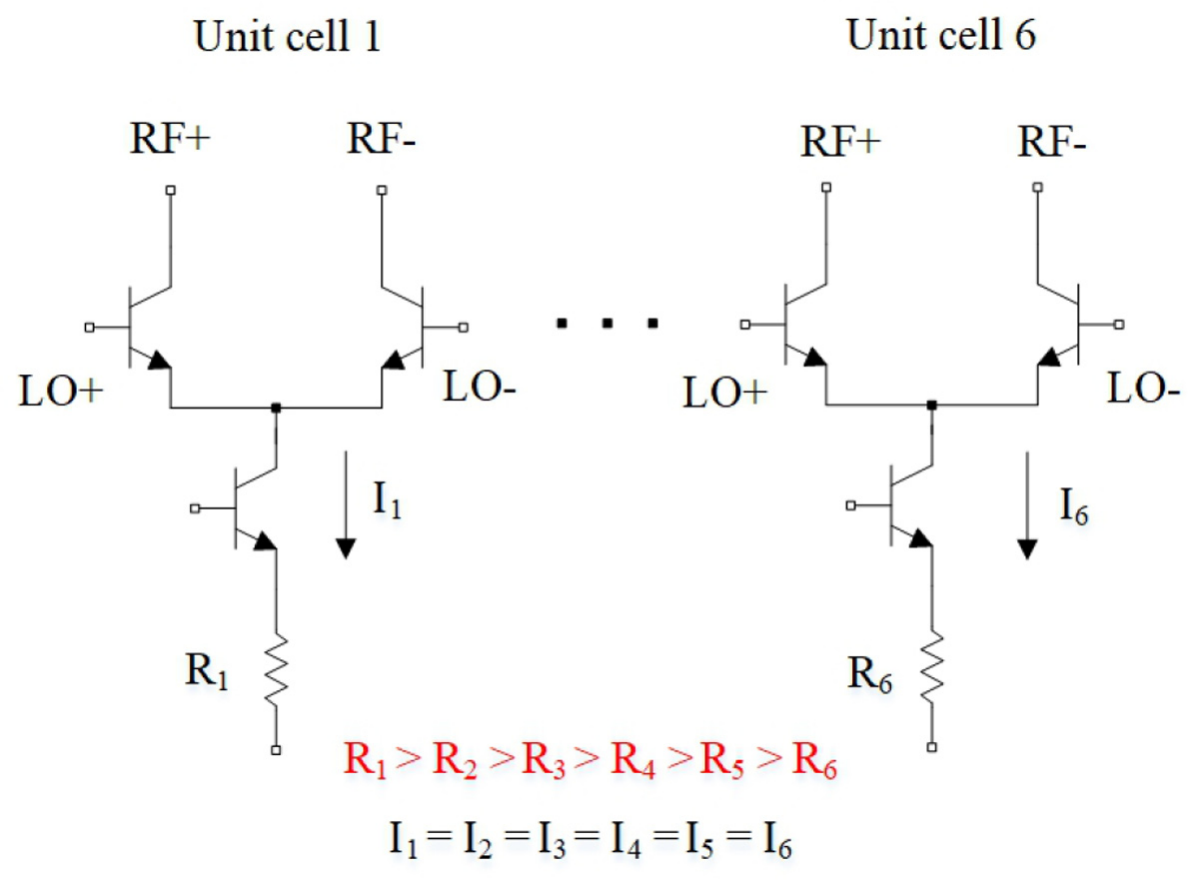
Main concept used for distributed mixer. RF, Radio Frequency; LO, Local Oscillator. ©2020 IEEE. Adapted from 125 with permission.

The novel distributed mixer, achieving the record bandwidth of 194 GHz is shown in work ^125^ ( Figure 20). As a method for liquidating the conversion gain degradations the use of non-equal emitter resistors among each cell was applied. Hence, the current of each unit cell can be adjusted separately to compensate for its non-uniformity.

Performance of the recently presented devices is shown in the Table 1.

**Table 1.** mmWave mixers. CG, Conversion Gain; LO, Local Oscillator; P _1 *dB*_, 1-dB Compression Point; P _*DC*_, DC power consumption.

	Frequency, GHz	CG, dB	LO power, dBm	Input P _1 *dB*_, dBm	P _*DC*_, mW	Area, mm ^2^
122	75-82	4.8	0	0.8	1.5	0.065
123	110-170	32	–2	–41	65	0.2
124	5-50	–15.5	15	-	0	1.05
125	0-194	–3	2	-	125	0.8
127	92-95	8.5	7.7	–12	11.5	0.33
128	180-194	–12.4	2	-	0	0.388
128	171-220	1	–4	–11	6.3	0.21

### 5.5 Phase shifters

Designing a phase shifter, achieving full 360-degree range, becomes a challenging task at mmWave frequencies. Since the phase shifter is a key block in transceivers with an RF-beamforming scheme, it has a large influence on the overall transceiver performance.

The fully passive solutions provide the best linearity, however, due to very high losses, these are almost not used at mmWave frequencies. One of the most common configurations for high frequency applications is a vector modulator (VM), which is especially suitable for the systems without strict linearity requirements. This solution allows to reduce losses or even to achieve a certain gain, while keeping the power consumption relatively low.

**Figure 21.**
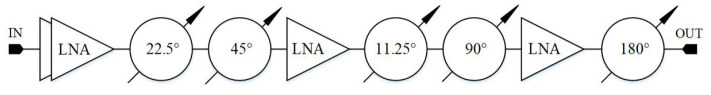
Integration with Low Noise Amplifier (LNA) stages. ©2018 IEEE. Adapted from 126 with permission.

There were several attempts to integrate LNA with phase shifter, such as ^126^ ( Figure 21). Here, the LNA stages were introduced between passive structures, performing partial phase shift.

The circuit provides 18 dB of gain, relatively low gain and phase errors (2 dB and 3.5 degrees). However, the linearity is quite poor, i.e. the input P _1 *dB*_ is -25 dBm.

**Figure 22.**
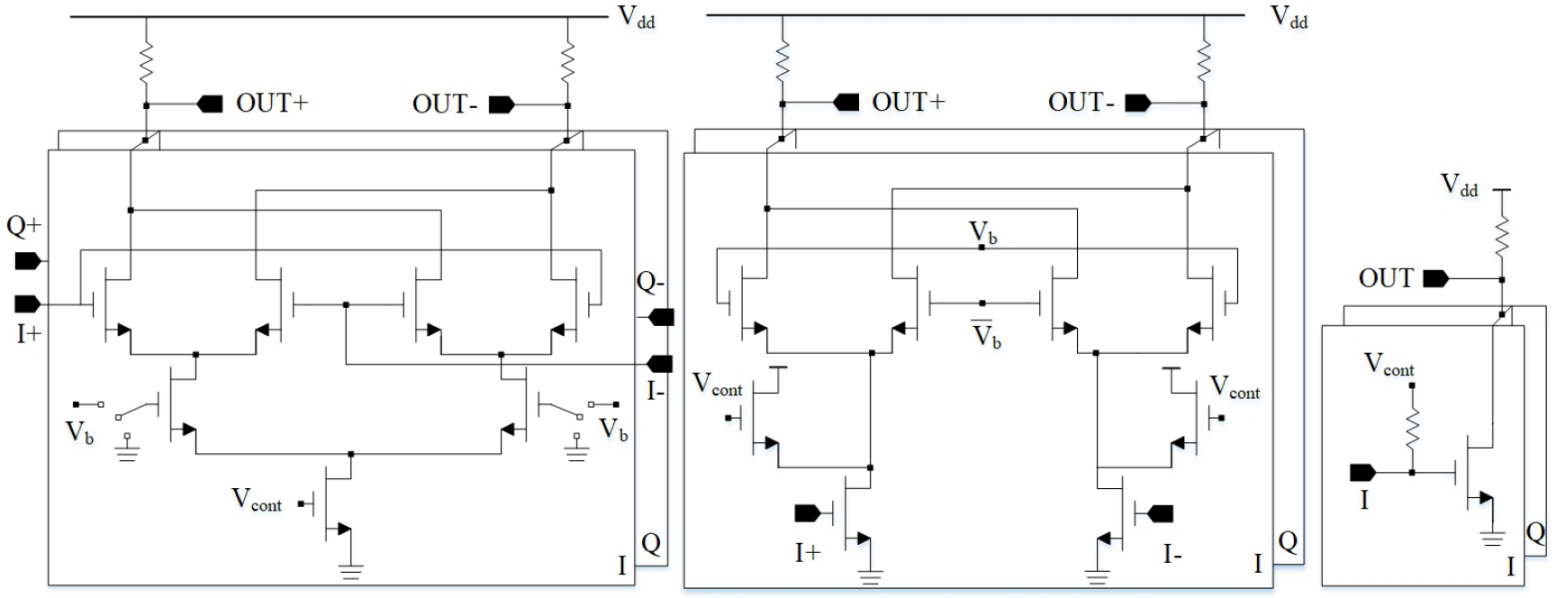
Variable-gain amplifier (VGA) architectures.

For the conventional VM, there are several approaches to build Variable-Gain Amplifiers (VGAs). The most well-known approach is to vary the transistor biasing. In this case, the Gilbert cell structure, cascode current steering structure, and the common source structure can be used ^129^ ( Figure 22).

The main disadvantage is that the bias variations lead to impedance variations, additional gain and phase errors.

**Figure 23.**
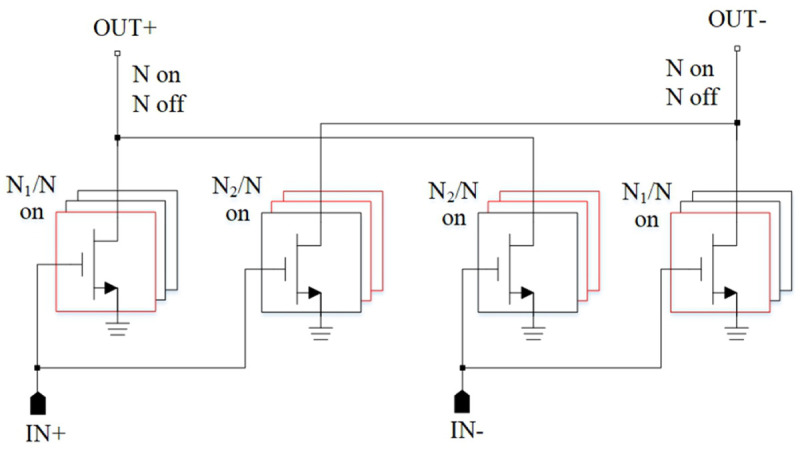
Variation of transistor width to build Variable-Gain Amplifier (VGA). ©2020 IEEE. Adapted from 129 with permission.

Another approach is to vary the transistor width to control the gain, as presented in 129 ( Figure 23). However, this increases the number of transistor connections which can have a significant impact at higher frequencies.

Important advantage of VMs is reduced size in comparison to fully passive configurations. This can be crucial, especially for large phased arrays. Some of the most area efficient examples are ^130– 132^. The phase shifter, presented in 130, occupies only 0.07 mm ^2^ of area.

Each application, however, dictates different requirements. In some cases, there are relatively tough specifications in terms of linearity, which leads to the need of finding trade-off between meeting this requirement and power consumption.

Some of the recent works are shown in the Table 2.

**Table 2.** mmWave phase shifters. P _*DC*_, DC power consumption.

	Frequency, GHz	Phase resolution, bits	Gain, dB	RMS gain error, dB	RMS phase error, degrees	Input P _1 *dB*_, dBm	P _*DC*_, mW	Area, mm ^2^
131	57.7-84.2	5	–6	0.8-1.1	2.7-1.1	5	17	0.11
129	51-66.3	5	–3.8	0.25-0.72	3-7	–0.23	5	0.3
132	78.8-92.8	4	–2.3	*<* 2	*<* 11.9	–7	21.6	0.17
137	94	12	–1.5	0.36	*<* 0.77	–5	-	-
130	160-190	4	–6.2	*<*1	*<*8	–13.5	12.4	0.07
138	71-84	6	7	0.46-0.76	1.35-3.5	–10	60	0.31

### 5.6 Low noise amplifier review

Low noise amplifier (LNA) is typically the first active device in any receiver system. The main role of the amplifier is to gain a weak input signal with a minimum additional noise contribution so that it can be used for the next processing in the system. Noise figure of the LNA therefore directly limits the sensitivity of the receiver because it can overlap the level of the input signal. If the performance of the LNA is insufficient, the rest of the receiver design and channel management to achieve necessary requirements will be useless.

**Figure 24.**
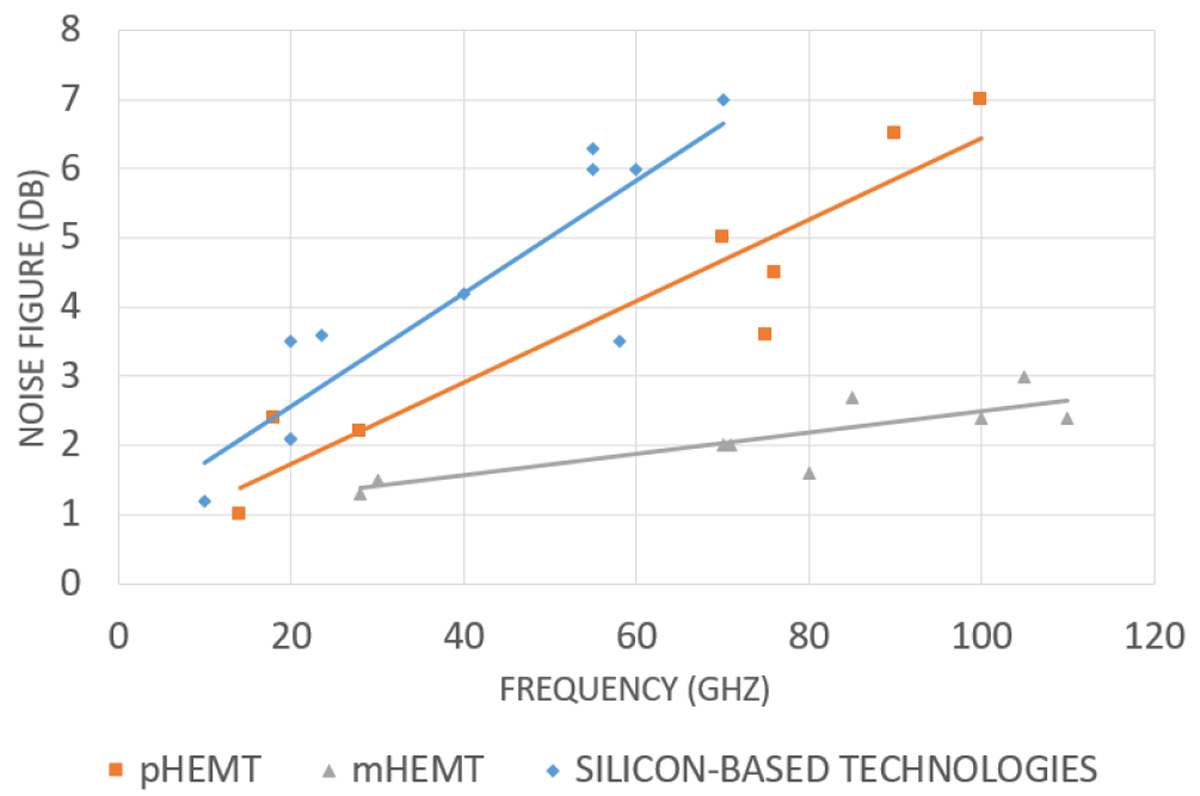
Comparison of noise figure for various technologies based on the data in Table 3. pHEMT, Pseudomorphic High Electron Mobility Transistor; mHEMT, Metamorphic High Electron Mobility Transistor.

The critical properties for all low noise transistor technologies is an electron high mobility in the channel ^133^. Such devised have low noise values because the variation of the current in the bandgaps is low compared to standard technologies. The most widely using transistor technology for the LNA today are Pseudomorphic High Electron Mobility Transistor (pHEMT) and Metamorphic High Electron Mobility Transistor (mHEMT). These technologies use an extremely thin layer of one of the semiconductors materials – so thin that the crystal lattice simply stretches to fit the other material or special buffer layer between two substrates to avoid electron traps ^134^. These techniques allow the construction of transistors with larger bandgap differences than otherwise possible, giving them better electrical performance ^135^. Table 3 summarizes state-of-the-art commercial and published LNAs. Figure 24 presents noise trend lines from the information in Table 3. Indium phosphide (InP) has some of the lowest noise figure and highest frequency performance ^136^, but this technology is more expensive and less frequently used for MMICs than GaAs HEMTs. Therefore, the InP technology is not presented in the Figure 24.

**Table 3.** Summary of the state-of-art mmWave Low Noise Amplifiers (LNAs).

LNA name/reference	Technology	Frequency of interest, GHz	Gain, dB	Noise figure, dB
CGY2125AUH/C1 OMMIC		14	25	1
ADL5725 Analog Devices		18	25.1	2.4
HMC1040LP3CE Analog Devices		28	23	2.2
ADL7003 Analog Devices		70	14	5
HMC8325 Analog Devices		75	21	3.6
CHA1077a98F UMS		76	15	4.5
CHA1008-99F UMS		90	17	6.5
CHA1008-99F UMS		100	17	7
CGY2128UH/C2 OMMIC		28	24	1.3
CGY2122XUH/C2 Ommic		30	32	1.5
CGY2260UH/C1 OMMIC		30	24	1.5
139		70	26	2
140		71	27	2
141		80	25	1.6
CGY2190UH/C2 OMMIC		85	23	2.7
140		100	20	2.4
CGY2190UH/C2 OMMIC		105	23	3
139		110	22	2.4
142	SiGe HBT	10	22	1.2
142	SiGe HBT	20	18	2.1
143	SiGe BiCMOS	20	32.8	3.5
144	CMOS SOI	20	19.5	2.1
145	CMOS	23.5	20	3.6
146	CMOS	40	14	4.2
147	BiCMOS	55	20	6
146	CMOS	55	15	6.3
148	SiGe	58	15	3.5
149	SiGe BiCMOS	60	32	6
147	BiCMOS	70	23	7

New processes based on SiGe, SOI CMOS, and GaAs materials have become increasingly popular for LNA manufacturing due to the improvement in performance is can offer in comparison to standard silicon counterpart. The mHEMP transistor technology is one of the better choice for the mmWave LNA from a noise minimization point of view, however, not only noise figure is important for the good mmWave receiver design. Due to the higher modulation schemes and channel characteristics envisioned for beyond 5G, improved distortion performance is required in the analog circuitry. Responsible for that parameter is amplifier linearity can be controlled on schematic design level. LNA linearity strongly depends on its power consumption ^150^. Using feed-forward and derivative superposition, post distortion and negative feedback linearization technics presented in 151 possible to get better tradeoff between linearity and amplifier current consumption.

### 5.7 Power amplifiers

The overall performance of a mmWave transmitter depends on the performance of the PA used therein ^152– 155^, given that the PA governs the transmitted power level, efficiency, circuit bandwidth, and linearity of a transmitter. Consequently, for best performance, the PA is required to deliver a specified output power while maintaining a high gain, a high power-added efficiency (PAE), and high linearity over a wideband of frequencies ^152– 155^. In MIMO systems, the specification of the PA in terms of output power depends on the beamforming technique adopted in the transmitter. Although the circuit specifications of the PA are not clearly defined yet, Shakib *et al*. ^152^ investigated the optimum output power, gain, and linearity of a PA deployed in a MIMO system at mmWave considering array scale, path loss, and other factors. Based on this investigation, the specifications of a 5G PA were presented in 156 and are summarized in Table 4.

**Table 4.** An example of a mmWave power amplifier specifications for 5G. ACPR, Adjacent-Channel Leakage Ratio; EVM, Error-Vector Magnitude; QAM, Quadrature Amplitude Modulation.

Frequency (GHz)	24.75-27.5 / 37-42.5
Bandwidth (MHz)	800
Saturated output power (dBm)	25 (III-V) / 17 (Si)
Average efficiency (%)	20
ACPR (dBc)	*−*27.5
EVM (%)	5 (64 QAM) or *<* 3 (256 QAM)

Furthermore, the output power and gain of a PA are directly related to the transistor technology adopted in the design thereof ^156, 157^. Hence, the first step in the design of a mmWave PA is the selection of the appropriate transistor technology.

***5.7.1 Technologies*.** The output power and gain of a PA are respectively related to the power density, cut-off frequency ( *f _T_*), and maximum oscillation frequency ( *f _MAX_*) of the transistor technology adopted in the design of the PA. Consequently, these factors constitute the main figure of merit (FoM) for technology comparison ^157^. Also, the beamforming technique adopted in the transmitter plays a crucial role in the selection of the PA transistor technology. The III-V compound semiconductor technologies such as gallium arsenide (GaAs) and gallium nitride (GaN), exhibit a high power density, gain, and efficiency over a broad frequency range.

**Figure 25.**
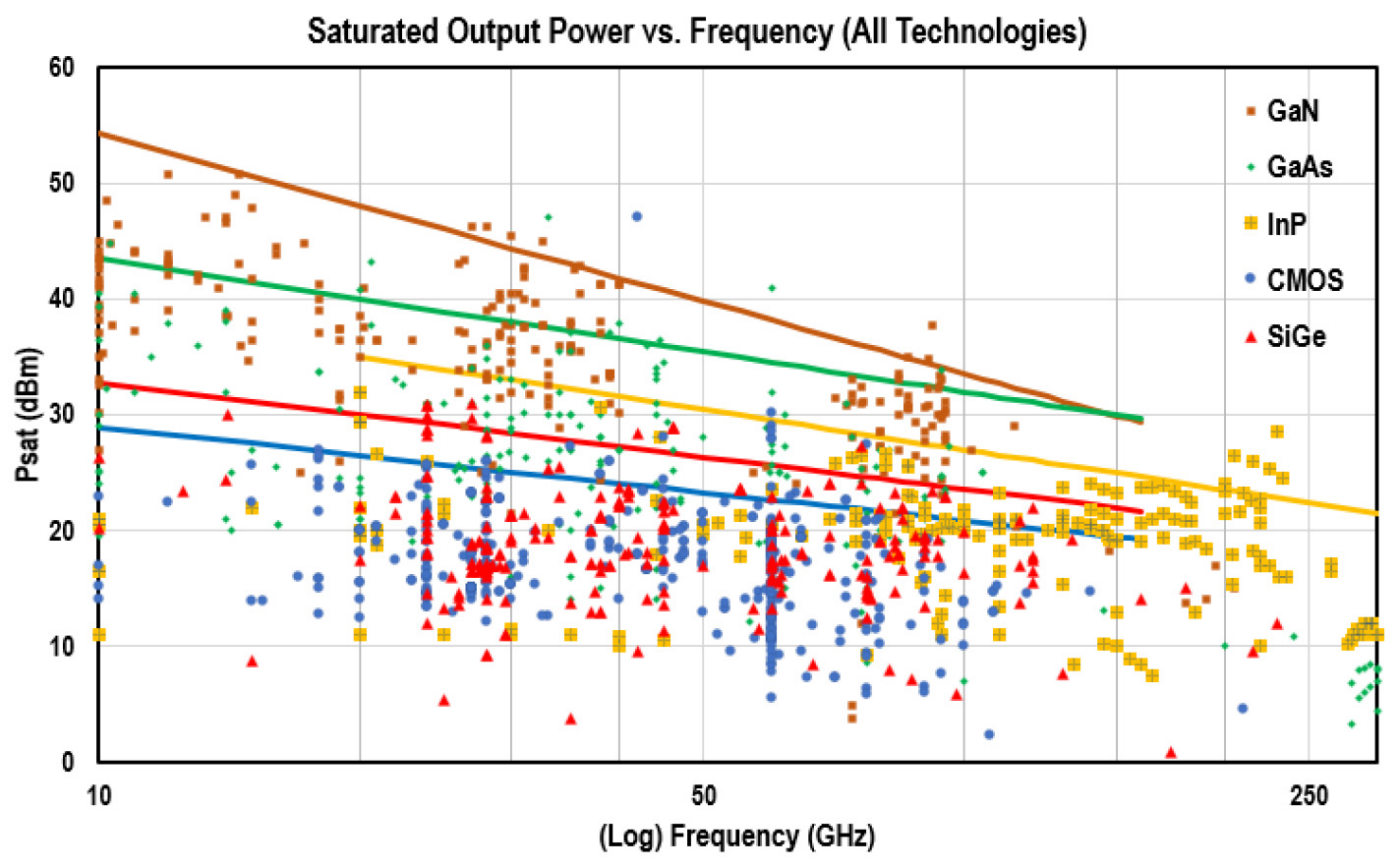
Saturated output power versus frequency for different transistors technologies. Own figure, utilising data published in the free access database ^158^.

While the silicon-based technologies such as complementary metal-oxide-semiconductor (CMOS) and BiCMOS SiGe (Silicon-Germanium) technologies offer a high degree of integration and low cost when mass-produced ^157^. The performance of PAs reported in the literature at mmWave frequencies for different transistors technologies is shown in Figure 25, extracted from the survey conducted at Georgia Tech ^158^. A comprehensive comparison between the process parameters of different transistor technologies is provided in [ Table 1, 158]. Transistor technologies with a higher power density (GaN or GaAs) are preferable in the case of analog beamforming given that a large number of antennas are fed by a single PA. While in the case of digital beamforming, transistor technologies (CMOS and SiGe) with a lower power density than GaN and GaAs but with a high degree of integration can be adopted since each antenna is fed by its own PA.

Regardless of the transistor technology used, it is difficult for a single-ended PA to deliver the specified output power and gain at mmWave frequencies. Moreover, the efficiency of the PA is limited by some characteristics of the transistor such as the ratio between the drain bias and the knee voltage ^157^. Also, the parasitic elements such as the output capacitance of the transistor have a detrimental effect on the efficiency and the achievable circuit bandwidth of the PA ^152– 157^. Hence, it is crucial to adopt appropriate solutions to maintain a high-efficiency operation over a broad frequency range. In addition to the power density of the transistor technology, the achievable output power relies on the breakdown voltage of the technology. III-V compound semiconductors such as GaN have a higher breakdown voltage than Si-based technologies. A low breakdown voltage implies that Si-based PAs are operated with a low supply voltage (hence low output power) to increase their reliability. The limitations associated with the low break down of Si-based technology such as CMOS are further exacerbated by the scaling down thereof.

***5.7.2 Design techniques*.** In an attempt to meet the output power requirement of a mmWave PA, on-chip power combining techniques such as the parallel connection of transistors, differential drive plus output balun and the series transformer combining techniques have been used in 159– 165. However, the loss of power combiner will reduce the PA’s gain and PAE. Furthermore, additional parasitic paths added by the extra transistors limit the PA’s performance. In the case of the differentially driven devices, the impedance transformation loss in the balun transformer limits the improvement in saturated output power to 2–3 dB while this improvement is limited to 2–2.5 dB in the case of multi-winding magnetic transformers due to the combiner size and mismatch losses ^152^. In an attempt to improve back-off efficiency, power amplifiers implemented using advanced topologies such as Doherty PA and envelop tracking (ET) PA have been reported in 166– 171 and 172– 174 respectively. The advantages and limitations of each are discussed in 154 alongside others topologies such as out-phasing and traveling-wave (distributed) PAs ^175^, with the latter aimed at improving the bandwidth rather than the efficiency. The results of recently published work on mmWave PAs are summarized in Table 5. Other PAs with similar performances have been reported in 157.

**Table 5.** Summary of the state-of-art mmWave power amplifiers. P _*SAT*_, Saturation Output Power; PAE _*SAT*_, Power Added Efficiency at Saturation; PAE _*OBO*_, Power Added Efficieny at -6dB back-off; SS Gain, Small-Signal Gain.

Ref.	Year	Technolo	f (GHz)	Topology	N.of stages	P _*SAT*_ (dB)	PAE _*SAT*_ (%)	PAE _*OBO*_ (%)	SS Gain (dB)
176	2014	40 nm GaN HEMT	65-110	Single-ended	3	31.1	27	-	20
159	2018	100 nm GaN/Si HEMT	37-43	Combined	3	40	23	-	18
160	2012	150 nm GaAs pHEMT	17-35	Combined	1	22.5	30	-	10
161	2003	100 nm GaAs mHEMT	32	4 ways Combined	2	35	40	-	24
162	2015	100 nm GaAs pHEMT	71-76	8-way combined	4	28	13	-	26
163	2019	45 nm CMOS SOI	56-63	24-way combined	3	28.5	15	-	24
164	2015	40 nm CMOS	71-86	4-way combined	2	20.9	22	-	18.1
175	2018	130 nm SiGe HBT	20-28	Distributed	4	19	8	-	-
165	2020	45 nm CMOS SOI	24-40	Differential cascode	2	19	36.6	-	12
177	2018	50 nm GaAs mHEMT	65-125	Stacked	1	22	10.7	-	16.8
178	2015	130 nm SiGe BiCMOS	93	Class-E	2	17.7	40.4	-	15
179	2018	45 nm CMOS	23.5-41	Continuous class F/F-1	-	18.6	45.7	-	11.4
166	2019	45 nm CMOS SOI	27	Cascode, mixed-signal Doherty	2	23.3	40.1	33.1	19.1
167	2019	45 nm CMOS SOI	60	Differential Doherty	3	20.5	26	16.6	13
168	2019	150 nm GaAs pHEMTs	26.5-29.5	Combined Doherty	2	25	35	25	10
169	2019	100 nm GaN/Si HEMT	27.5-28.35	Single device Doherty	2	32	25	28	10
170	2015	40 nm CMOS	72	Doherty	-	21	13.6	7	18.5
171	2018	45 nm CMOS SOI	65	Doherty	-	19.4	27.5	20.1	12.5

## 6 Conclusions

With the viewpoint on increasing data rates, efficiency and cellular coverage, novel integrated millimetre wave solutions, signal processing techniques, and network architectures are investigated. An overview of distributed massive MIMO communication systems and architectures is provided in Section 2. Synchronization and radio structure simplicity, make the centralized processing with analogue radio over fibre and attractive option. Signal processing challenges in DM-MIMO are discussed in Section 2, including synchronization and calibration, precoding and channel estimation, signal compression and quantization, multiple access techniques, system characterization and simulation methods. The joint design of channel estimation, transmit precoding, and network architecture tailored to mmWave massive MIMO systems is well worth future research effort. Signal compression/quantization is critical to alleviating the impact of fronthaul constraints on spectral efficiency (SE) and energy efficiency (EE) performance. The multiple access techniques under consideration include non-orthogonal multiple access and random access, while initial access remains an open issue. It is clear that many challenges still exist in the characterization and multi-physics simulation of the future of MIMO systems. The radio frequency front-end components are considered in Section 4 and Section 5. For transceiver systems, a move towards antenna-on-chip and antenna-in-package solutions is probable, but many challenges for the rest of the transceiver in terms of stability, power distribution and management, synchronization and control would need to be addressed.

## Data availability

No data are associated with this article.

## Ethics and consent

Ethical approval and consent were not required.
