# Supplementary material for: The state of the art in beyond 5G distributed massive multiple-input multiple-output communication system solutions
Source: Open Res Eur. 2022 Sep 2;2:106. [Version 1] doi: 10.12688/openreseurope.14501.1 (PMC10654493; doi:10.12688/openreseurope.14501.1)
Supplement: Peer review history

# Reviewer report: Reviewer response for version 1

**DOI:** 10.21956/openreseurope.15654.r33904

**Published:** 2023-11-16

**Authors:** Rashmi Sinha, Santosh Kumar Mahto

**Affiliations:** 1 Department of Electronics and Communication Engineering,, National Institute of Technology Jamshedpur, Jamshedpur, Jharkhand, India; 2 Indian Institute of Information Technology Ranchi, Ranchi, Jharkhand, India

The author reported the state of the art in beyond 5G distributed massive multiple-input multiple-output communication system solutions.

 The overview and challenges of distributed massive MIMO communication systems and network architectures are discussed. Also, an overview of the state of the art in millimetre wave antennas and electronics is presented. The following questions should be addressed:

1. Are soft computing techniques utilized to estimate the channel in DM-MIMO?
2. What are the design challenges involved in millimetre wave antennas and electronics?
3. What is mutual coupling? What are the different techniques used to minimize mutual coupling?
4. What is OTFS? Different methods used for delay Doppler channel estimation in OTFS should be discussed in the manuscript.

Is the review written in accessible language?

Yes

Are all factual statements correct and adequately supported by citations?

Yes

Are the conclusions drawn appropriate in the context of the current research literature?

Partly

Is the topic of the review discussed comprehensively in the context of the current literature?

Partly

Reviewer Expertise:

Different methods used for delay Doppler channel estimation in OTFS should be discussed.

We confirm that we have read this submission and believe that we have an appropriate level of expertise to confirm that it is of an acceptable scientific standard, however we have significant reservations, as outlined above.

# Reviewer report: Reviewer response for version 1

**DOI:** 10.21956/openreseurope.15654.r34537

**Published:** 2023-11-16

**Authors:** Daniyal Ali Sehrai

**Affiliations:** 1 University of Oviedo, Gijon, Spain

This review article presents an outlook on 5G distributed massive MIMO communication systems, the signal processing, characterization, and simulation challenges, and an overview of the state of the art in millimeter wave antennas and electronics. The communication system is discussed regarding challenges and the possible solutions for the successful deployment of the beyond-5G communication system. A few suggestions are given below which can make this review article more insightful for the readers:

- As for 5G communication, two critical chunks of spectrum, i.e., Sub-6 GHz and mm-wave targeted. *(This should be little be highlighted in the introduction section)*
- Below 6 GHz, some other frequency bands are already allocated for many different applications like 5.35-5.69 GHz (LTE 46/WLAN). So, why is still Sub-6 GHz targeted for 5G and what were the drawbacks? *(This question should also be addressed in the introduction section)*
- Is Beyond-5G only focused on THz frequency bands? *(Please put some discussion about it in the introduction section)*
- Will the transmission based on the antennas operating at THz frequency bands be harmful to human tissues? What are the possible safety measures?
- Due to higher data rates and capacity demands, each new communication generation is targeting the higher part of the spectrum which causes a small coverage area and many base station deployments to have a large coverage area. *(Please discuss the deployment, maintenance, and operation cost complexity as compared to the previous technologies)*
- At THz frequencies, the antenna size will become quite small, which will also pose some fabrication challenges. *(Please put some discussion and possible solutions)*
- As in the case of mm-wave transmission, atmospheric attenuations are a quite severe challenge. For this, high-gain antennas are a possible solution with MIMO capabilities. What about THz frequencies? For the THz-based antenna transmission, what would be the possible gain requirements?​
- I suggest replacing figure 15 with its colored copy (RGB) instead of the black in white one.
- I would also suggest adding a comparison table in section 4, where authors can put a comparison from third generation to beyond 5G in terms of frequency bands targeted/aimed, pros and cons, challenges, and possible solutions (brief key statements would be enough), and the data rates achieved/aimed.

Is the review written in accessible language?

Yes

Are all factual statements correct and adequately supported by citations?

Yes

Are the conclusions drawn appropriate in the context of the current research literature?

Yes

Is the topic of the review discussed comprehensively in the context of the current literature?

Partly

Reviewer Expertise:

RF Sensors and Circuits, Antennas, Metamaterials, Implanted Antennas, Electromagnetic Imaging, Radars, Electromagnetics, RF front end, MIMO, Arrays

I confirm that I have read this submission and believe that I have an appropriate level of expertise to confirm that it is of an acceptable scientific standard, however I have significant reservations, as outlined above.

# Reviewer report: Reviewer response for version 1

**DOI:** 10.21956/openreseurope.15654.r33908

**Published:** 2023-11-16

**Authors:** Jagadeesh Babu kamili

**Affiliations:** 1 St. Ann's College of Engineering & Technology, Chirala, Andhra Pradesh, India

Overall, the manuscript is presented in a lucid and effective manner and hence can be accepted after incorporating the following minor suggestions:

  

- On page 4, second column, second paragraph, in the sentence, " ***However, because all the antennas are co-located the line of sight (LOS) conditions between base station and user cause high correlation among various users and proves to be a bottleneck for a system throughout*** **…"**   Please check whether it is throughout or throughput.
- In Section 4, regarding antenna design and array scaling, the authors have presented the impact and role of antenna design in 5G and beyond in a lucid manner. However, the main problem in antenna design when employed in MIMO systems is mutual coupling, which deteriorates the overall system performance. Hence, the authors are suggested to give some information related to the mutual coupling issue in 5G MIMO.
- Authors are asked to give some information related to sub 6 GHz 5G systems.
- Regarding reference [88], on page no. 14, the authors mentioned that the use of cavity-backed antennas is required to improve radiation efficiency. However, the antenna design mentioned in Reference [88] does not use cavity back technology. 
- Similarly, as mentioned by the authors, References [90] and [91] are not related to metamaterials.
- As a large number of references are given, authors are advised to check the accuracy of all the citations given in the main manuscript part and References part.

Is the review written in accessible language?

Yes

Are all factual statements correct and adequately supported by citations?

Partly

Are the conclusions drawn appropriate in the context of the current research literature?

Yes

Is the topic of the review discussed comprehensively in the context of the current literature?

Yes

Reviewer Expertise:

Antenna design for MIMO and 5G communications.

I confirm that I have read this submission and believe that I have an appropriate level of expertise to confirm that it is of an acceptable scientific standard.
